# Plac1^+^ Tumor Cell‐Treg Interplay Supports Tumorigenesis and Progression of Head and Neck Cancer

**DOI:** 10.1002/advs.202417312

**Published:** 2025-03-08

**Authors:** Xiaoyan Meng, Zhonglong Liu, Lu Zhao, Ran Li, Luoman Gan, Liren Cao, Jingjing Sun, Lingfang Zhang, Yue He

**Affiliations:** ^1^ Department of Oral Maxillofacial & Head and Neck Oncology Shanghai Ninth People's Hospital Shanghai Jiao Tong University School of Medicine College of Stomatology Shanghai Jiao Tong University National Center for Stomatology National Clinical Research Center for Oral Diseases Shanghai Key Laboratory of Stomatology Shanghai 200011 P. R. China; ^2^ Department of Oral Pathology Shanghai Ninth People's Hospital Shanghai Jiao Tong University School of Medicine College of Stomatology Shanghai Jiao Tong University National Center for Stomatology National Clinical Research Center for Oral Diseases Shanghai Key Laboratory of Stomatology Shanghai 200011 P. R. China; ^3^ Suzhou Lingdian Biotechnology Co., Ltd Suzhou 215000 P. R. China

**Keywords:** cancer/testis antigens, epithelial growth factor receptor, head and neck squamous cell carcinoma, placenta‐specific protein 1, tumor immunity, tumor microenvironment

## Abstract

Cancer/testis antigen (CTA) family is restricted to germline and tumor cells and plays an important role during cancer initiation and progression. Five single‐cell and two bulk RNA‐seq datasets are integrated to screen genes in the CTAs family, revealing that Placenta specific protein 1 (Plac1) is specifically expressed in head and neck squamous cell carcinoma (HNSCC) cells. Sp1 Transcription (*SP1)* is identified as a specific regulator of *Plac1*, which is confirmed by cleavage under targets and tagmentation (CUT&Tag)‐seq. With in vitro experiments, in vivo subcutaneous tumor, and a transgenic autochthonous tumor model, it is revealed that *Plac1* expression promotes HNSCC progression by inducing epidermal growth factor receptor endocytosis and recycling to increase PI3K/AKT signaling pathway activity. Then, it is revealed that *Plac1^+^* tumor cells recruit CD4^+^ T cells via CXCL11/CXCR3 and induce Treg differentiation via PVR/TIGIT, which in turn activates the tumorigenic signaling of *Plac1^+^* tumor cells via LTA/LTBR and forms a reciprocal protumor loop. These findings provide insights into molecular features of CTAs in HNSCC and facilitate the development of personalized treatment strategies.

## Graphical abstract

Meng et al. screen genes in the CTAs family and find Plac1 is specifically expressed in head and neck squamous cell carcinoma (HNSCC) cells. They reveal that Plac1 expression promotes HNSCC progression directly by activating PI3K/AKT signaling pathway. In the meantime, Plac1^+^ tumor cells shape a Treg‐related immunosuppressive microenvironment via CXCL11/CXCR3 and PVR/TIGIT, which in turn activates the tumorigenic signaling of Plac1+ tumor cells via LTA/LTBR and forms a reciprocal protumor loop. These findings provide insights into molecular features of CTAs in HNSCC and facilitate the development of personalized treatment strategies.

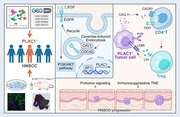

## 1 Introduction

Head and neck squamous cell carcinoma (HNSCC), with 1 000 000 new cases annually, is the sixth most common cancer worldwide.^[^ ^1^, ^2^ ^]^ Despite considerable progress in therapeutic approaches for HNSCC, particularly with the introduction and development of target therapy and immunotherapy, the prognosis remains unfavorable for a substantial number of patients, especially patients with advanced HNSCC with tumor relapse or lymph node/distant metastasis.^[^ ^3^ ^]^ The major challenge for HNSCC treatment lies in tumor heterogeneity, including tumor cell heterogeneity, tumor microenvironment (TME) heterogeneity, and individual heterogeneity, which highlights the need for a deeper understanding of the molecular mechanisms of HNSCC initiation and progression.

Cancer/testis antigens (CTAs) are an antigen family whose expression is restricted to germline cells and tumor cells.^[^ ^4^ ^]^ Emerging evidence shows that CTAs participate in tumorigenic processes and are implicated in multiple hallmarks of cancer, including increased cell viability and proliferation.^[^ ^5^, ^6^ ^]^ A series of clinical trials of immunotherapies targeting these genes have been conducted in patients with melanoma, lung cancer, and other solid tumors.^[^ ^7^ ^]^ However, in the context of HNSCC, several unresolved problems remain: Are HNSCC‐specific CTAs present? How do CTAs participate in the tumorigenesis of HNSCC? How does CTA expression influence the whole TME?

The study of heterogeneous cell populations has been greatly enhanced by the development of single‐cell RNA sequencing (scRNA‐seq), which allows comprehensive investigation of the transcriptomic profiles of individual cell populations.^[^ ^8^ ^]^ This technique has been employed to study the TME in various cancer types, including HNSCC.^[^ ^9^, ^10^, ^11^, ^12^, ^13^ ^]^ Moreover, the novel techenique spatial transcriptomics (ST) has overcome the limitation of scRNA‐seq (lacks spatial relationship information) by directly obtaining transcriptomic expression profiles at a high resolution close to the single‐cell level.^[^ ^14^ ^]^ The abundant public scRNA‐seq datasets and ST data from HNSCC patients have served as valuable resources for us to conduct our research.

In this study, we performed integrative bulk RNA‐seq and scRNA‐seq data analysis of HNSCC and revealed a special CTA, Placenta specific protein 1 (*Plac1*), is highly expressed in HNSCC cancer cells. *Plac1* is a gene encoding a transmembrane antigen that plays a significant role in trophoblast proliferation and has been associated with the progression of cancers.^[^ ^15^ ^]^ According to the previous reports, *Plac1* is mainly expressed in the trophoblasts in the placenta, which exhibit an invasive growth pattern to absorb nutrition to support embryogenesis. In the context of cancer growth, *Plac1* has been identified with protumor characteristics in several cancers such as cervical cancer and breast cancer.^[^ ^16^, ^17^ ^]^ However, these studies were restricted in the phenotypic validation in the in vitro level without further mechanistic investigation. Moreover, whether the expression of *Plac1* on tumor cells addresses impacts on the whole TME remains to be clarified.

In the current study, tumorigenic *Plac1* ^+^ tumor cells were demonstrated to enhance protumor signaling pathways and to accelerate tumor growth in in vitro and in vivo HNSCC models. Moreover, through ST analysis, multiplex immunofluorescence (mIF) staining, and experimental validation, we revealed that *Plac1* ^+^ tumor cells generate a Treg‐related immunosuppressive TME, which in turn activates tumorigenic signaling in *Plac1* ^+^ tumor cells; this then forms a reciprocal loop for HNSCC progression. These findings may provide more insights into the molecular features of CTAs in HNSCC and may help facilitate the development of personalized treatment strategies.

## 2 Results

### 2.1 Overview of the Expression Pattern of *Plac1* as an HNSCC‐Specific CTA

**Figure 1:**
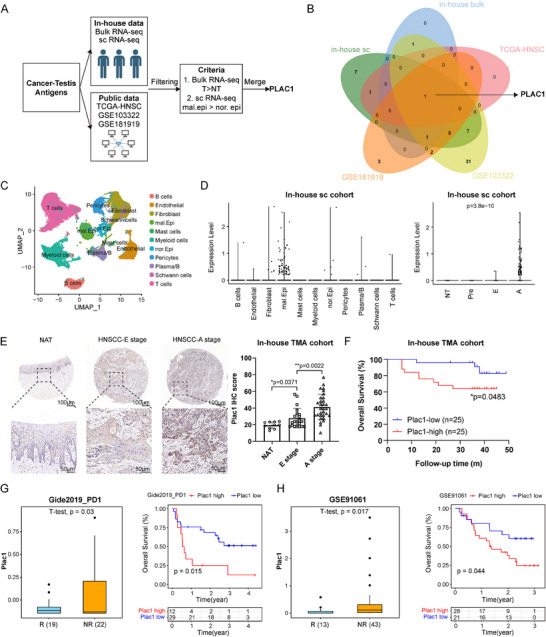
An overview of *Plac1* expression pattern in HNSCC. A,B) Schematic representation (A) and Venn plot (B) of the CTAs screening pipeline (created with bioRender.com). C) Uniform Manifold Approximation and Projection (UMAP) plots of different cell types from in‐house scRNA‐seq data. D) Violin plots show expression levels of *Plac1* of different cell types from in‐house scRNA data. E) Representative IHC staining (left) and quantitative results (right) of Plac1 expression in different HNSCC stages (NAT: n = 10, E: n = 21, A: n = 31). Scale bar, 100 and 50 µm. F) Kaplan–Meier curves show patients with higher Plac*1* expression levels exhibit poorer OS in the in‐house TMA cohort. G,H) Box plots show *Plac1* expression levels in R and NR groups (left) and the Kaplan‐Meier curves show patients with higher *Plac1* expression levels exhibit poorer OS in ICI cohorts (right). Panel (G) shows results of Gide2019 cohort (n = 41) and (H) shows results of GSE91061 (left: n = 56, right: n = 49). *P* values were calculated by one‐way ANOVA in D, by two‐sided Student's *t*‐test in panels (E, G, H), and by two‐sided log‐rank test in panels (F, G, H). *p < 0.05, **p < 0.01.

CTAs have been widely reported to be expressed in different types of cancer.^[^ ^7^, ^18^ ^]^ In the present study, we focused on HNSCC and established a screening strategy that integrates multiple bulk RNA‐seq and scRNA‐seq datasets to obtain CTAs that are highly expressed in HNSCC cells (**Figure** **1**A). For the bulk RNA‐seq datasets, both in‐house cohort and public TCGA‐HNSC cohort were included, and we selected CTAs whose expression levels were significantly higher in HNSCC tumors than those in normal tissue. Regarding scRNA‐seq, we referred to the in‐house cohort^[^ ^13^ ^]^ and two public cohorts (GSE103322^[^ ^9^ ^]^ and GSE181919^[^ ^12^ ^]^) and selected CTAs whose expression levels were specifically higher in malignant epithelial cells than those in normal epithelial cells. After intersecting the CTA gene candidates in each dataset with the above criteria, we obtained only one gene, *Plac1*, which is highly expressed in HNSCC tumor cells, for further research (Figure 1B).

As shown by the bulk RNA‐seq data (Figure S1A,B, Supporting Information), *Plac1* was more highly expressed in tumor tissues (T) than in normal tissues (NT), which reflects the common expression pattern of CTAs. Analysis in the single‐cell resolution revealed that *Plac1* expression was restricted to malignant epithelial cells (Figure 1C,D, Figure S1C,D, Supporting Information). We also referred to two scRNA‐seq datasets without malignant identification information (Figure S1E,F, Supporting Information, GSE185965^[^ ^19^ ^]^ and GSE188737^[^ ^20^ ^]^) and we observed that *Plac1* expression was specific to epithelial cells. Moreover, the in‐house scRNA‐seq cohort included HNSCC tumor samples representing different clinical stages^[^ ^13^ ^]^ and *Plac1* was significant more highly expressed in malignant epithelial cells from A‐stage samples (Figure 1D). As for lymph node metastasis and tumor relapse, we found that compared to primary lesions, epithelial cells in metastatic lesions exhibited higher *Plac1* expression level (*p* < 0.0001 both in GSE182227^[^ ^21^ ^]^ and GSE188737^[^ ^20^ ^]^) (Figure S1G, Supporting Information) and compared to primary tumors, recurrent tumors showed higher *Plac1* expression level (*p* = 0.038 in GSE173855^[^ ^22^ ^]^) (Figure S1H, Supporting Information). These results indicated that *Plac1* was associated with advanced HNSCC status. The ST samples were also analyzed to investigate *Plac1* expression pattern in HNSCC^[^ ^23^ ^]^ and we found that *Plac1* expression was restricted in the tumor cells (Figure S1I,K, Supporting Information).

We subsequently validated the expression pattern of Plac1 in an in‐house tumor tissue microarray (TMA) cohort, which included normal oral mucosa and HNSCC tissues from different stages, as reported in our previous publication (Figure S2A, Supporting Information).^[^ ^13^ ^]^ The Plac1 expression level was found to be associated with advanced tumor status according to multiple criteria. According to the American Joint Committee on Cancer (AJCC) staging manual, cancers can generally be staged according to tumor (T), lymph node (N), and metastasis (M) status. When the samples were categorized according to the clinical stage, the Plac1 Immunohistochemistry (IHC) score was higher in E‐stage tissues than in normal adjacent tissues (NAT) (*p =* 0.0371) and higher in A‐stage tumors than in E‐stage tumors (*p* = 0.0022) (Figure 1E). Similarly, the percentages of clinical stage III and IV (defined as clinical A stage) (*p* = 0.0253) and pathological T3 and T4 (pT3 and pT4, defined as pathological A stage) (*p* = 0.0481) patients were greater in the Plac1‐high group than those in the Plac1‐low group (Figure S2B, Supporting Information). In addition, the Plac1 IHC score was greater in T3/4 samples than that in T1/2 samples when divided by T stage (*p* = 0.0024) and greater in the N2/3 samples than that in the N0/1 samples when divided by N stage (*p* = 0.0344) (Figure S2C, Supporting Information). Notably, we collected tumor bulk (TB) and invasive tumor frontiers (ITF) regions from the same patient and found that the Plac1 expression level was greater in ITF regions than that in TB regions (*p* = 0.0004) (Figure S2D, Supporting Information), which suggests the potential invasive characteristics of *Plac1* ^+^ malignant epithelial cells.

The above results indicated that *Plac1* expression was associated with advanced clinical stages and malignant characteristics of HNSCC. Moreover, *Plac1* expression showed important prognostic significance: In the in‐house TMA cohort, patients with higher *Plac1* expression levels showed shorter overall survival (OS) (*p* = 0.0483) (Figure 1F), which was consistent with our previous study.^[^ ^24^ ^]^ For advanced HNSCC patients, immune checkpoint inhibitor (ICI) therapy is one of the most important therapeutics. Interestingly, *Plac1* expression was related to poor response and survival in ICI cohorts. We analyzed four public datasets (Gide2019_PD1,^[^ ^25^ ^]^ GSE91061,^[^ ^26^ ^]^ Nathanson2017,^[^ ^27^ ^]^ and IMvigor210^[^ ^28^ ^]^) and patients were divided into response (R) and nonresponse (NR) groups according to their response to therapy, after which the expression levels of *Plac1* were determined. Higher *Plac1* expression was detected in the NR group (Gide2019_PD1: *p* = 0.03, GSE91061: *p* = 0.017, Nathanson2017: *p* = 0.038) (Figure 1G,H, Figure S2E, Supporting Information). Moreover, patients with higher *Plac1* expression had poorer OS than their counterparts with lower *Plac1* expression (Gide2019_PD1: *p* = 0.015, GSE91061: *p* = 0.044, IMvigor210: *p* = 0.025) (Figure 1G,H, Figure S2F, Supporting Information). These results showed the clinical prognostic significance of *Plac1* as a specific CTA gene of HNSCC.

Finally, for further experiments, we validated *Plac1* expression in HNSCC cell lines. The *Plac1* expression level was highly heterogeneous among HNSCC cell lines and was significantly higher in HNSCC cells than that in normal epithelial cells (NC) (Figure S3A–C, Supporting Information). Among the HNSCC cell lines evaluated, *Plac1* was relatively highly expressed in HN6 cells and expressed at low levels in Cal27 cells. Therefore, we selected these two cell lines for the following gene‐editing experiments.

In summary, we identified *Plac1* as an HNSCC‐specific CTA whose expression is restricted to HNSCC tumor cells and is associated with advanced tumor stages and poor clinical outcomes.

### 2.2 Transcription Factor *SP1* Induces *Plac1* Expression During HNSCC Initiation and Progression

According to previous reports, in addition to its expression in tumor tissues, *Plac1* is also highly expressed in the placenta and is downregulated during embryonic development and maturation.^[^ ^29^ ^]^ The process by which *Plac1* expression is downregulated during embryogenesis is exactly the opposite of that by which *Plac1* is upregulated during tumor initiation and progression. Therefore, we wondered whether the regulators of *Plac1* are similar in these two processes; we then sought to identify the key drivers of *Plac1* and HNSCC progression through the analysis of scRNA‐seq embryogenesis data.

**Figure 2:**
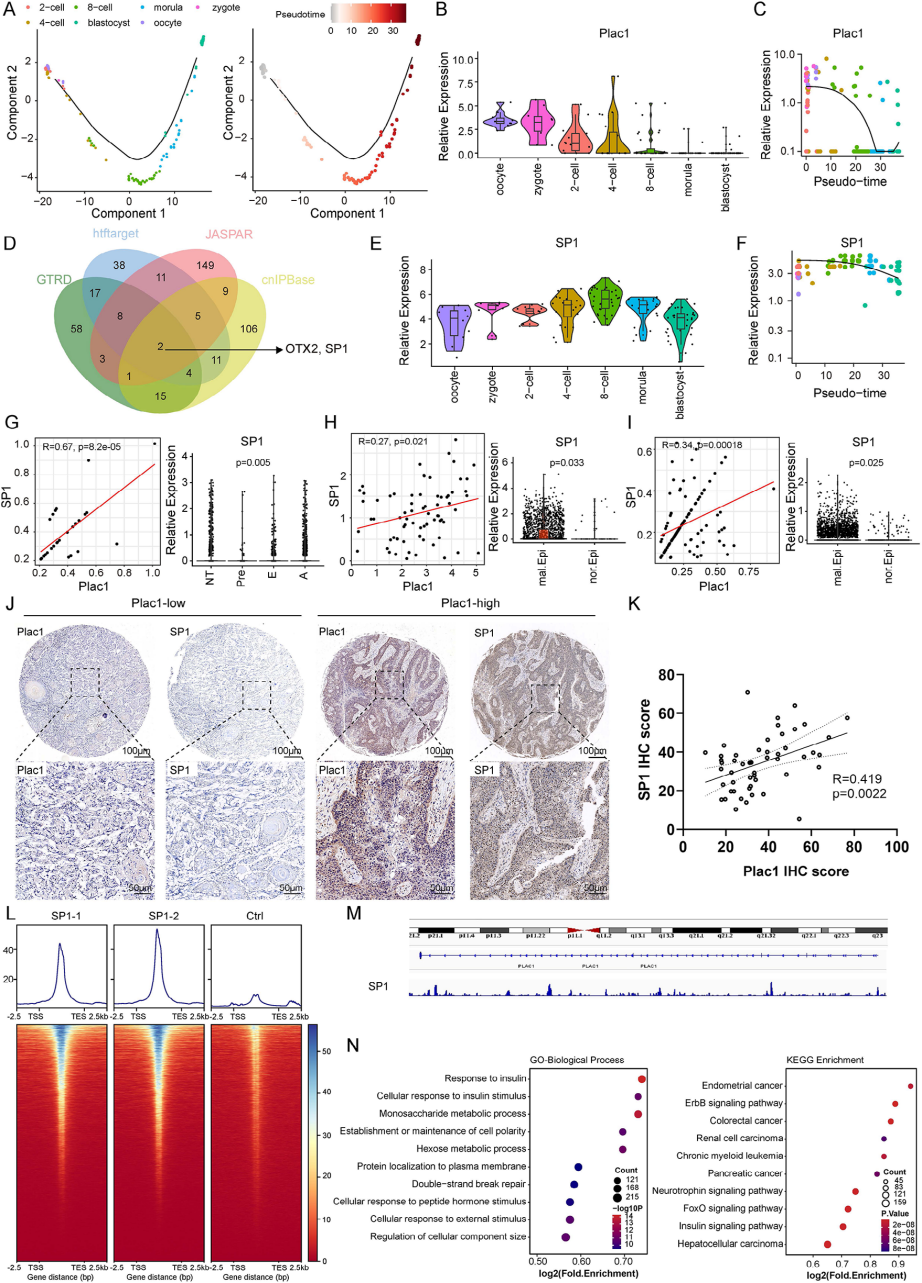
Transcription factor *SP1* induces *Plac1* expression during HNSCC initiation and progression. A) Potential trajectory of epithelial cells during embryonic development inferred by Monocle2. B,C) The expression level of *Plac1* in different stages during embryonic development (B) and alongside the pseudotime axis (C). (D) Venn diagram shows overlapping of potential TFs that regulate *Plac1* expression. E,F) The expression level of *SP1* in different stages during embryonic development (E) and alongside the pseudotime axis (F). G–I) Scatter plots show the Pearson correlation between expression of *Plac1* and *SP1* in the in‐house cohort (G), GSE103322 (H), and GSE181919 (I). Violin plots of *SP1* expression level in malignant and normal epithelial cells are shown in the right panel. J) Representative IHC staining of SP1 and Plac1. Scale bar, 100 µm and 50 µm. (K) Pearson correlation of IHC scores of Plac1 and SP1, related to (J) (n = 52). L) Heatmap shows the genomic occupancy of *SP1* in HN6 cells. M) Analysis of SP1‐occupied *Plac1* gene loci signatures. N) GO (left) and KEGG pathway (right) enrichment of SP1‐marked genes, related to (L). *P* values were calculated by one‐way ANOVA in G, by one‐sided Wilcoxon test in H, I, and by hypergeometric test in N.

Here, we examined three public scRNA‐seq datasets of human embryogenesis (GSE36552,^[^ ^30^ ^]^ GSE44183,^[^ ^31^ ^]^ and GSE71317) and extracted epithelial cells from them, including epithelial cells at the 2‐cell stage, 4‐cell stage, 8‐cell stage, as well as in the blastocyst, morula, oocyte, and zygote stages (**Figure** **2**A). We constructed an epithelial development trajectory with these epithelial cells using Monocle2 and found that the cell distribution exhibited satisfactory consistency with the embryonic development stages and *Plac1* expression was downregulated during embryogenesis and along the pseudotime axis, further supporting our hypothesis (Figure 2B,C).

Then, we performed in silico analysis via four public transcription factor (TF) databases (GTRD,^[^ ^32^ ^]^ htftarget,^[^ ^33^ ^]^ JASPAR,^[^ ^34^ ^]^ and chIPBase^[^ ^35^ ^]^) and identified two candidate TFs, Orthodenticle Homeobox 2 (*OTX2)* and Sp1 Transcription Factor (*SP1)*, for *Plac1* (Figure 2D). Next, we analyzed the expression trends of these TFs in embryogenesis data and determined the correlation between their expression levels and Plac1 expression levels in the in‐house and public scRNA‐seq cohort. Both *OTX2* and *SP1* tended to be downregulated overall during embryogenesis, which aligns with the expression trend of *Plac1* during embryonic development (Figure 2E,F, Figure S4A,B, Supporting Information). However, the expression levels of *OTX2* were not significantly different among different stages, and no significant difference was observed between malignant and normal epithelial cells (Figure S4C,D, Supporting Information). In contrast, the expression level of *SP1* was significantly greater in malignant cells than in normal cells (in‐house data: *p* = 0.005, GSE103322: *p* = 0.033, GSE181919: *p* = 0.025) and was greater in tumor samples of advanced‐stage HNSCC (in‐house data: *p* = 0.005). Moreover, *SP1* expression was found to be positively correlated with *Plac1* expression in all three scRNA‐seq datasets (in‐house data: R = 0.67, *p* < 0.0001; GSE103322: R = 0.27, *p* = 0.021; GSE181919: R = 0.34, *p* = 0.00018) (Figure 2G,I). In addition, an IHC assay was performed to validate the *SP1* expression pattern in clinical tumor tissues and we observed a robust positive correlation between SP1 IHC scores and plac1 IHC scores (R = 0.419, *p* = 0.0022), which further supported our hypothesis that *SP1* could induce *Plac1* expression during HNSCC initiation and progression (Figure 2J,K).

According to the above results, we further validated our hypothesis in HNSCC cells. We knocked down *SP1* in HN6 and Cal27 cells using small interfering RNAs (siRNA) and found that both the mRNA and protein expression levels of Plac1 were downregulated in the SP1‐KD groups (Figure S5A,B, Supporting Information). To determine how *SP1* regulates *Plac1* expression and HNSCC progression, we performed a cleavage under targets and tagmentation (CUT&Tag) assay using an anti‐SP1 antibody. As shown in Figure 2L, compared with the negative control group, the *SP1* group presented significant peaks. Notably, the peaks were identified in the transcriptional start site region of the *Plac1* gene (Figure 2M), which indicates the regulatory effect of *SP1* on *Plac1*. Gene Ontology (GO) and Kyoto Encyclopedia of Genes and Genomes (KEGG) analysis of the SP1‐related peaks revealed relevant enrichment of the ErbB signaling pathway, FoxO signaling pathway, establishment or maintenance of cell polarity, and several cancer‐related pathways (Figure 2N). The protumor effects of *SP1* were consistent with those of *Plac1*, which was further validated by in vitro migration and invasion assays (Figure S5C, Supporting Information). In summary, these results suggest that, like its role in the embryonic microenvironment, *SP1* might be involved in the expression of *Plac1* during tumor initiation and progression.

### 2.3 *Plac1* Expression in Epithelial Cells Promotes HNSCC Initiation and Progression

**Figure 3:**
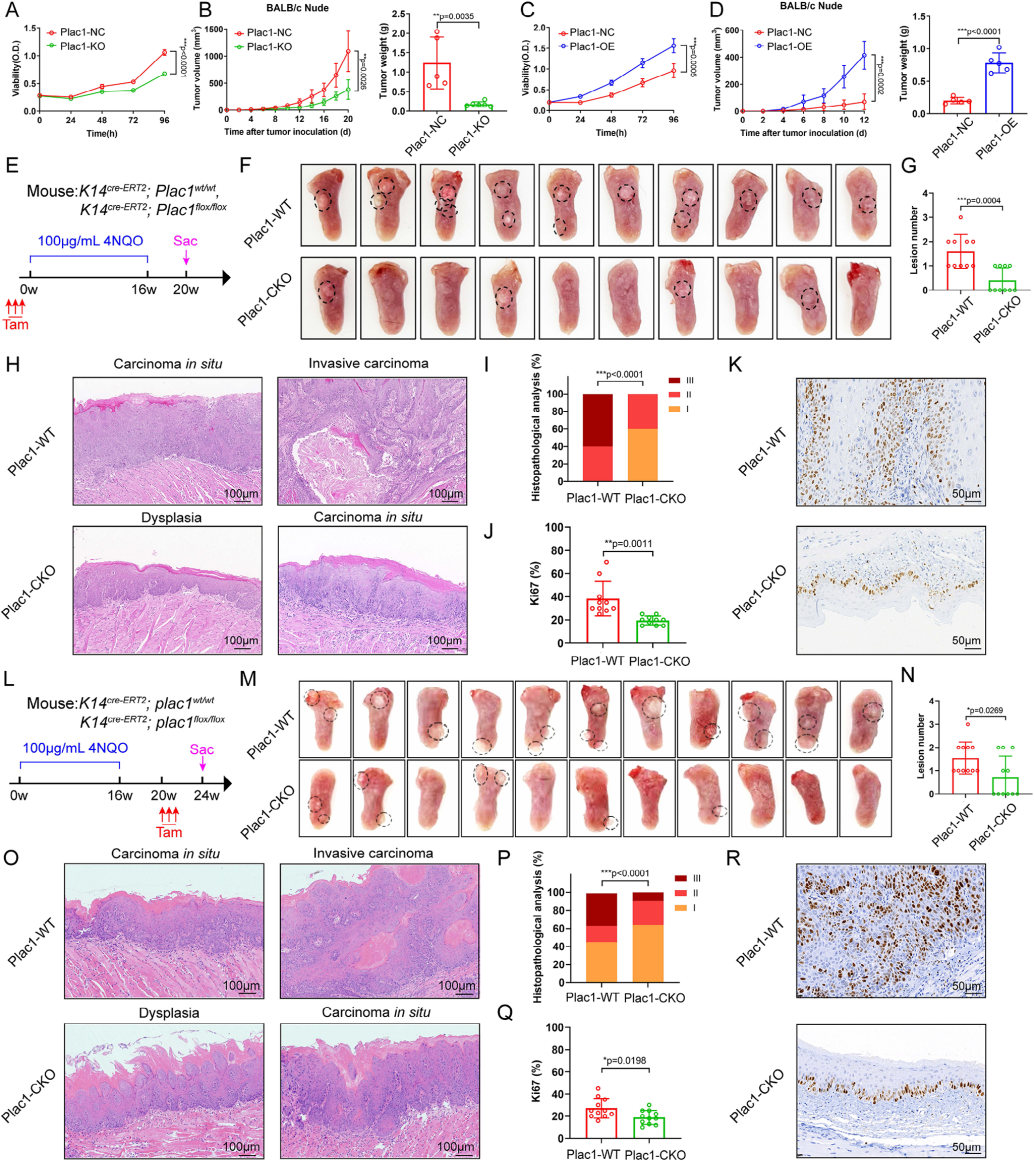
*Plac1* expression in epithelial cells promotes HNSCC initiation and progression. A,C) Cell viability of HNSCC cells with *Plac1* KO (A) and OE (C) (n = 5). B,D) Tumor volumes (left) and tumor weights (right) after injection of HNSCC cell lines with *Plac1* KO (B) and OE (D) (B: NC: n = 5, KO: n = 6; D: n = 5). E) Schematic diagram for inducing mouse HNSCC and tamoxifen induction of *Plac1* deletion in epithelial cells. Tamoxifen was administered at 0 week. Three consecutive injections were intraperitoneally given to reach the best efficiency of *Plac1* deletion. F–I) Gross photos (F, lesions are indicated with circles), quantification of lesions (G), representative H&E staining (H), and histopathological analysis (I) of tongue tissues of Plac1‐WT and Plac1‐CKO mice, related to (E) (n = 10). Scale bar = 100 µm. J,K) Quantification (J) and representative Ki67 IHC staining (K) of tongue tissues of Plac1‐WT and Plac1‐CKO mice, related to (E) (n = 10). Scale bar = 50 µm. L) Schematic diagram for inducing mouse HNSCC and tamoxifen induction of *Plac1* deletion in epithelial cells. Tamoxifen was administered at 20^th^ week. Three consecutive injections were intraperitoneally given to reach the best efficiency of *Plac1* deletion. M–P) Gross photos (M, lesions are indicated with circles), quantification of lesions (N), representative H&E staining (O), and histopathological analysis (P) of tongue tissues of Plac1‐WT and Plac1‐CKO mice, related to (L) (n = 11). Scale bar = 100 µm. Q,R) Quantification (Q) and representative Ki67 IHC staining (R) of tongue tissues of Plac1‐WT and Plac1‐CKO mice, related to (L) (n = 11). Scale bar = 50 µm. *P* values were calculated by two‐sided Student's *t*‐test in A‐D, G, J, N, Q, and were calculated by Chi‐square test in I, P. *p < 0.05, **p < 0.01, ***p < 0.001.

Next, we investigated the protumor effects of *Plac1* in HNSCC. Based on the background expression level of *Plac1* (Figure S3B,C, Supporting Information), we selected HN6 cells to generate a Plac1‐knockout (KO) cell line via the CRISPR/cas9 system, and Cal27 cells to generate a Plac1‐overexpressing (OE) cell line via a tagged plasmid (Figure S6A,B, Supporting Information). Through CCK8 and Annexin V/PI assays, we found that *Plac1* expression directly enhanced the proliferation and inhibited the apoptosis activity of HNSCC cells (**Figure** **3**A,C, Figure S6C–F, Supporting Information). To evaluate tumor growth ability in vivo without interruption of immune effects, HNSCC cells were injected subcutaneously into Balb/c nude mice which lack a mature lymphoid immune system. As expected, HN6‐Plac1‐KO tumors were smaller (*p* = 0.0026) and weighed less (*p* = 0.0035) than HN6‐Plac1‐NC tumors (Figure 3B), while Cal27‐Plac1‐OE tumors were larger (*p* = 0.0002) and weighed more (*p* < 0.0001) (Figure 3D). These results suggested that *Plac1* expression endow epithelial cells with malignant characteristics in a cancer‐cell‐autonomous manner.

To simulate the original HNSCC initiation and progression process in vivo, immune competent mice (C57BL/6) were used for the autochthonous tumor model: 4‐Nitroquinoline (4NQO) was administrated in their drinking water (100 µg/mL) for 16 weeks at which point they received normal drinking water, as previously described.^[^ ^36^ ^]^ Consistent with previous reports,^[^ ^37^ ^]^ histopathological evaluation revealed that the mice developed various squamous lesions, including varying degrees of hyperplasia and squamous cell carcinomas (SCC) (Figure S7A,B, Supporting Information). To determine the effects of *Plac1* on epithelial cells in HNSCC, we crossed Keratin 14 (K14) Cre/ERT2 mice (K14^Cre‐ERT2^) with Plac1^f/f^ mice to generate K14^Cre‐ERT2^; Plac1^f/f^ or Plac1^f/‐^ (Plac1‐conditional knockout, Plac1‐CKO) and K14^Cre‐ERT2^; Plac1^W/W^ or Plac1^W/−^ (Plac1‐wildtype, Plac1‐WT) mice^[^ ^38^ ^]^ (Figure S7C,D, Supporting Information). Hematoxylin‐eosin (H&E) staining and IHC for Plac1 in major organ samples revealed that the conditional knockout of *Plac1* in epithelial cells resulted in no significant systemic toxicity (Figure S7E,F, Supporting Information). We then depleted *Plac1* before the mice were fed drinking water containing 4NQO (Figure 3E). Twenty weeks after 4NQO treatment, tongue lesion numbers and degree of tumor invasion were analyzed as previously described.^[^ ^39^ ^]^ Histological analysis revealed that epithelial‐cell‐specific deletion of *Plac1* significantly reduced the number of lesions (Figure 3F,G, *p* = 0.0004). For the degree of tumor invasion, dysplasia was identified as Grade I, carcinoma in situ was identified as Grade II, and invasive carcinoma was identified as Grade III. We found that the degree of tumor invasion was markedly alleviated by *Plac1* ablation, as confirmed by H&E staining (Figure 3H,I, *p* < 0.0001). Additionally, to account for the inhibition of HNSCC growth in Plac1‐CKO mice, we measured the epithelial cell proliferation ratio via Ki67 IHC staining. In Plac1‐CKO mice, proliferating cells were restricted mainly to the basal layer of the epithelium, whereas proliferating cells were widely distributed in dysplasia and tumor lesions in Plac1‐WT mice, which presented a greater cell proliferation ratio than Plac1‐CKO mice (Figure 3J,K, *p* = 0.0011).

According to the tumor inhibition effects of *Plac1* ablation, we were interested in whether *Plac1* deletion could inhibit HNSCC growth when *Plac1* expression was already increased in tumor cells. We treated the mice with tamoxifen 20 weeks after SCC induction, when the SCCs began to develop (Figure 3L). Four weeks after *Plac1* deletion, the number of tongue lesions was significantly lower in Plac1‐CKO mice (Figure 3M,N, *p* = 0.0269). Both the degree of invasion (*p* < 0.0001) and the ratio of proliferating tumor cell (*p* = 0.0198) were also significantly decreased in Plac1‐CKO mice (Figure 3O–R). The above results confirm that *Plac1* expression in epithelial cells is crucial for HNSCC initiation and progression.

### 2.4 *Plac1* Sensitizes the PI3K/AKT Pathway of HNSCC Cells by Promoting Caveolae‐Induced Epidermal Growth Factor Receptor Endocytosis and Recycling

**Figure 4:**
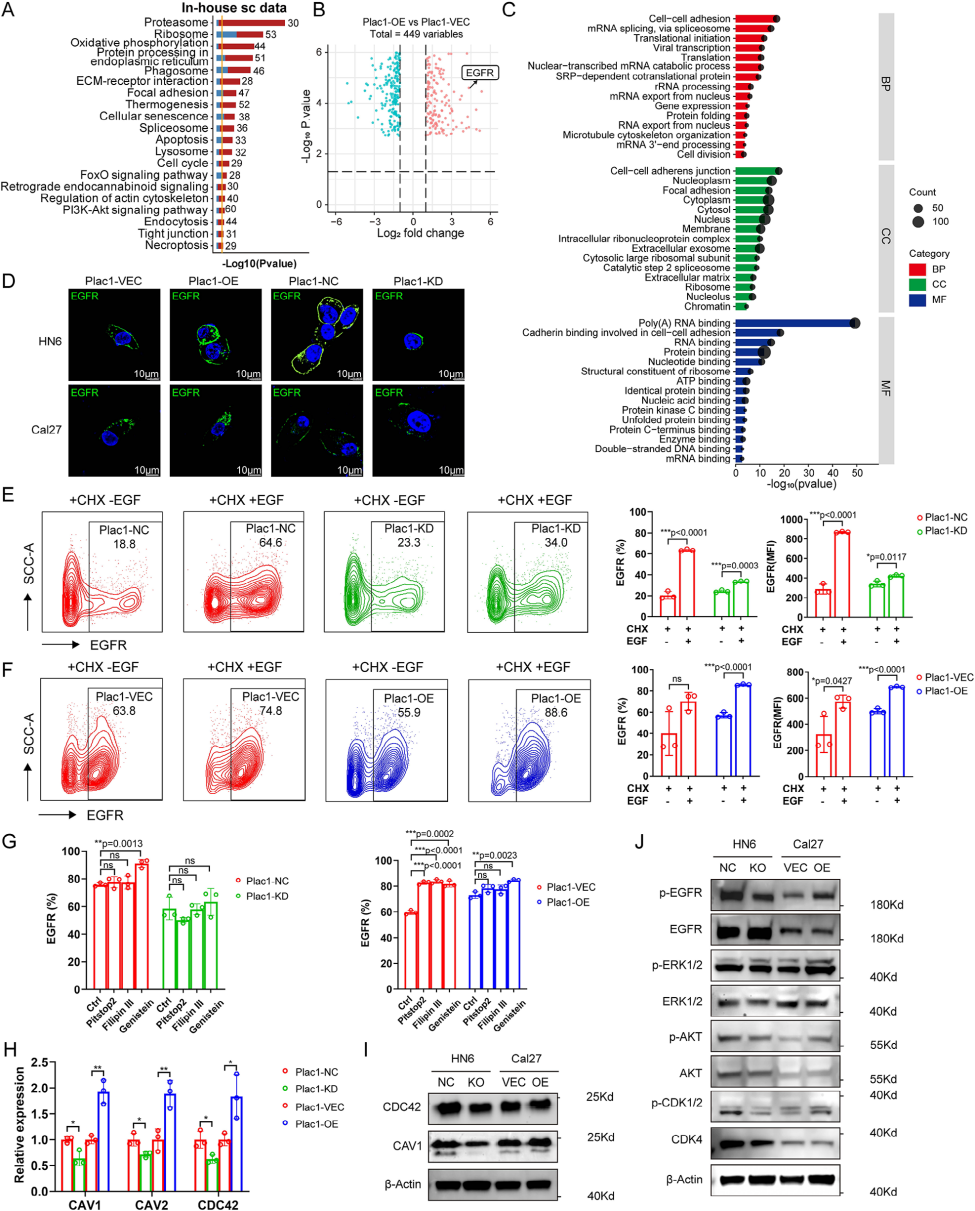
*Plac1* sensitizes the PI3K/AKT pathway of HNSCC cells by promoting caveolae‐induced EGFR endocytosis and recycling. A) KEGG pathway enrichment of DEGs between *Plac1* ^+^ and *Plac1^−^* malignant epithelial cells of in‐house scRNA‐seq cohort. B) Volcano plot shows differentially phosphorylated proteins between Plac1‐OE and Plac1‐VEC HNSCC cells. C) GO enrichment of differentially phosphorylated proteins between Plac1‐OE and Plac1‐VEC HNSCC cells, related to (B). D) Representative ICC images of EGFR of HNSCC cells from different groups. Green: EGFR, blue: Dapi. Scale bar = 10 µm. E,F) Representative flow cytometry images (left) and quantification results of EGFR^+^ cell ratio and MFI (right) of HNSCC cells with different treatments (n = 3). G) Quantification results of EGFR^+^ cell ratio of HNSCC cells treated with different endocytosis inhibitors (n = 3). H,I) mRNA (H) and protein (I) expression levels of genes related to caveolae‐induced endocytosis (n = 3). J) WB analysis of the levels of the PI3K/AKT pathway proteins of Plac1‐NC, Plac1‐KO, Plac1‐VEC, and Plac1‐OE HNSCC cells. *P* values were calculated by hypergeometric test in A, C, and by two‐sided Student's *t*‐test in E‐H. *p < 0.05, **p < 0.01, ***p < 0.001.

To explore the underlying mechanisms by which *Plac1* deletion ameliorated mouse tongue SCCs, we reanalyzed in‐house scRNA‐seq data and compared *Plac1* ^+^ and *Plac1* ^−^ epithelial cells in human HNSCC tissues. As shown by KEGG enrichment results, *Plac1* ^+^ epithelial cells presented significant upregulation of genes associated with proteasome, oxidative phosphorylation, ECM‐receptor interaction, focal adhesion, and cell cycle, which indicated their proliferative and invasive biological activities (**Figure** **4**A). KEGG enrichment results of differentially expressed genes (DEGs) comparing *Plac1^+^* epithelial cells to other epithelial cells of the ST samples showed similar pathways (e.g., Proteasome, Endocytosis, Focal adhesion, and Cell cycle) (Figure S1J, L, Supporting Information). In addition, we performed proteomics and phosphoproteomic analyses of Plac1‐OE and Plac1‐VEC cells to investigate the biological effects of *Plac1* expression at the protein level (Figure 4B). GO analysis revealed that Plac1‐OE cells exhibited significant increases in the phosphorylation level of proteins associated with cell‐cell adhesion, which was consistent with the scRNA‐seq results (Figure 4C). Notably, epidermal growth factor receptor (EGFR) was one of the most highly phosphorylated proteins in Plac1‐OE cells (Figure 4B). EGFR is a crucial oncogene in HNSCC,^[^ ^40^ ^]^ and has been reported to undergo endocytosis and recycling after EGF signaling activation. Interestingly, in both in‐house and public scRNA cohorts, we observed enrichment of endocytosis and PI3K‐AKT signaling pathway which were downstream pathways of EGFR activation, suggesting that *Plac1* is likely associated with EGFR activation and endocytosis (Figure 4A, Figure S8A–C, Supporting Information).

Using an immunocytometry (ICC) assay, we observed that after 5 min of EGF stimulation, fluorescence staining of EGFR was increased in the cytoplasm and was greater in Plac1‐OE cells than Plac1‐KD cells (Figure 4D). Cycloheximide (CHX) is an inhibitor for protein synthesis,^[^ ^41^ ^]^ and thus we pretreated cells with CHX to inhibit EGFR synthesis after EGF stimulation. Using this method, the EGFR expression level on the membrane could be mainly attributed to the endocytosis and recycling of resident EGFR. We found that EGF stimulation led to a significant increase in the EGFR expression level and EGFR^+^ cell ratio, indicating the receptor recycling process of EGFR. Moreover, ablation of *Plac1* alleviated this trend (EGFR^+^ percentage: Plac1‐NC: *p* < 0.0001, Plac1‐KD: *p* = 0.0003; EGFR mean fluorescent intensity (MFI): Plac1‐NC: *p* < 0.0001, Plac1‐KD: *p* = 0.0117) (Figure 4E). Consistent results were observed in Plac1‐VEC and Plac1‐OE cells (EGFR^+^ percentage: Plac1‐VEC: *p* > 0.05, Plac1‐OE: *p* < 0.0001; EGFR MFI: Plac1‐VEC: *p* = 0.0427, Plac1‐OE: *p* < 0.0001) (Figure 4F). These results showed that *Plac1* expression promoted EGFR endocytosis and recycling, whereas *Plac1* deletion inhibited this process. Endocytosis pathways are generally classified into clathrin‐mediated, caveolae‐mediated, and clathrin/caveolae‐independent endocytosis pathways.^[^ ^42^ ^]^ Therefore, to determine which pathway contributes most to *Plac1*‐related EGFR endocytosis in HNSCC cells, we pretreated cells with Pitstop2 (clathrin‐mediated pathway inhibitor),^[^ ^43^ ^]^ Filipin III (clathrin/caveolae‐independent endocytosis pathway inhibitor),^[^ ^44^ ^]^ and Genistein (caveolae‐mediated pathway inhibitor).^[^ ^44^ ^]^ Using flow cytometry, we found that only Genistein treatment significantly increased the percentage of EGFR^+^ cells (Plac1‐NC: *p* = 0.0013, Plac1‐VEC: *p* = 0.0002, Plac1‐OE: *p* = 0.0023), whereas Pitstop2 and Filipin III were not effective (Figure 4G, Figure S8D, Supporting Information), which suggested that *Plac1*‐related endocytosis is part of the caveolae‐mediated endocytosis pathway. Next, we investigated how *Plac1* expression promotes caveolae‐mediated endocytosis. Again, we referred to the scRNA‐seq data. By intersecting upregulated genes (*Plac1* ^+^ versus *Plac1* ^−^ malignant epithelial cells) identified in the “endocytosis” pathway from in‐house data and GSE103322, we obtained a list of genes that were associated with endocytosis and were specifically expressed in *Plac1^+^* tumor cells (Figure S8E, Supporting Information). Among them, *CAV1*,^[^ ^45^ ^]^ *CAV2*,^[^ ^46^ ^]^ and *CDC42* ^[^ ^47^ ^]^ attracted our attention as they encode crucial proteins that form caveolae complexes. We confirmed that *Plac1* expression was positively associated with CAV1, CAV2, and CDC42 expression at both mRNA and protein levels (Figure 4H‐I), which may contribute to the upregulation of endocytosis activities in *Plac1* ^+^ cells. The endocytosis and recycling of EGFR can maintain its activity and further promote downstream signaling pathways, including the PI3K/AKT pathway (Figure 4A). As shown in Figure 4J, Western Blotting (WB) analysis revealed that the phosphorylated protein levels of EGFR, ERK1/2, AKT, and CDK1/2 were significantly increased by *Plac1* overexpression and inhibited by *Plac1* knockout in HNSCC cells. In summary, we investigated protumor effects and mechanisms of *Plac1* in HNSCC cells, which promote EGFR endocytosis and recycling to enhance the PI3K/AKT signaling pathway for cell proliferation and invasion.

### 2.5 *Plac1* Is Associated with the Formation of the Immunosuppressive TME

**Figure 5:**
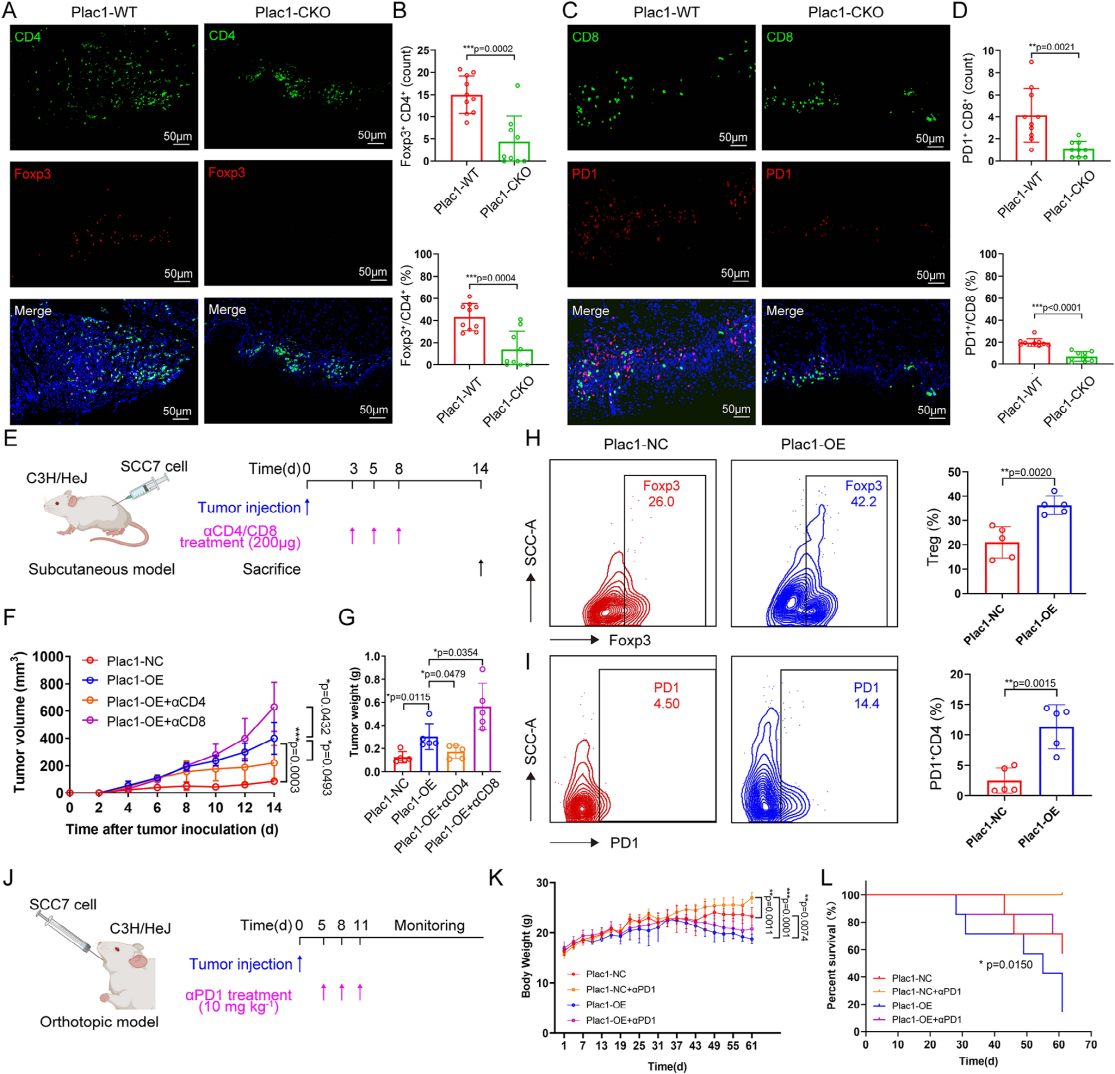
*Plac1* is associated with the formation of the immunosuppressive TME. A–D) Representative IF images (A,C) and quantification results (B,D) of Tregs (A,B) and CD8_Tex (C,D) of tongue tissues from Plac1‐WT and Plac1‐CKO mice (Plac1‐WT: n = 10, Plac1‐CKO: n = 9). Green: CD4 (A) or CD8 (C), red: Foxp3 (A) or PD1 (C), blue: Dapi. Scale bar = 50 µm. E) Schematic diagram of subcutaneous xenograft tumor model and αCD4/CD8 treatment (created with bioRender.com). F,G) Tumor volumes (F) and tumor weights (G) of different groups (n = 5). H,I) Representative flow cytometry images (left) and quantification results (right) of Treg ratio (H) and PD1^+^ CD4^+^ T cell ratio (I) of different groups (n = 5). J) Schematic diagram of orthotopic xenograft tumor model and αPD1 treatment (created with bioRender.com). K,L) Body weight curves (K) and survival curves (L) of different groups (n = 7). *P* values were calculated by two‐sided Student's *t*‐test in panels (B, D, F–I, K), and by two‐sided log‐rank test in panel (L). *p < 0.05, **p < 0.01, ***p < 0.001.

According to our previous study, *Plac1* is associated with the immunosuppressive microenvironment of HNSCC.^[^ ^24^ ^]^ To further investigate how *Plac1* expression affects the immune microenvironment, we performed immunofluorescence (IF) staining of Tregs and exhausted CD8^+^ T cells, two key immunosuppressive cells in tongue tissues of Plac1‐CKO and Plac1‐WT mice. We found that *Plac1* deletion significantly decreased both the number and the ratio of Treg cells and exhausted CD8^+^ T cells (**Figure** **5**A–D, Figure S9A–D, Supporting Information). Using a subcutaneous tumor xenograft model in immune‐competent mice (C3H/HeJ), we evaluated whether *Plac1* expression promoted HNSCC and shaped an immunosuppressive microenvironment in vivo (Figure 5E). Moreover, we injected mice HNSCC cells subcutaneously in immunodeficient mice (Balb/c nude) (Figure S9E,F, Supporting Information). In C3H/HeJ mice, Plac1‐OE cells formed larger tumors (*p* = 0.0003) and weighed more (*p* = 0.0115) than tumors derived from Plac1‐NC cells (Figure 5F,G) and we found that the tumor‐promoting effects were more significant in immune‐competent mice than those in immunodeficient mice (Balb/c nude: *p* = 0.0024, C3H/HeJ: *p* = 0.0003) (Figure S9G, Supporting Information), which indicated that the *Plac1*‐related immune microenvironment was crucial during HNSCC progression. Anti‐CD4 and anti‐CD8 neutralizing antibodies were administered to evaluate how CD4^+^ T or CD8^+^ T influenced the tumor‐promoting effects of *Plac1* expression (Figure 5E, Figure S9H–I, Supporting Information). As shown in Figure 5E–G, neutralizing CD8^+^ T cells enhanced the tumor‐promoting effects of Plac1‐OE cells (tumor volume: *p* = 0.0432, tumor weight: *p* = 0.0354), which suggested that the antitumor cytotoxicity of CD8^+^ T cells inhibited tumor growth. Interestingly, neutralization of CD4^+^ T cells in mice injected with Plac1‐OE cells reduced both tumor volume (*p* = 0.0493) and weight (*p* = 0.0479) to levels comparable to those in the Plac1‐NC group (both tumor volume and weight: ns between groups). These results indicated that CD4^+^ T cells play an important role in the mechanism of tumor‐promoting effects of *Plac1*. Moreover, we observed that although no significant difference was found in the exhausted CD8^+^ T cell or CD4^+^ T cell ratio between Plac1‐NC and Plac1‐OE groups, the ratios of Foxp3^+^ cells (*p* = 0.0020) and PD1^+^ cells (*p =* 0.0015) among CD4^+^ T cells were greater in Plac1‐OE groups (Figure 5H–I, Figure S9J–L, Supporting Information). Bulk RNA‐seq of tumors also revealed that DEGs in Plac1‐OE group were enriched in CD4^+^ T cell immunity pathways, such as antigen processing and presentation, Th1 and Th2 cell differentiation, and Th17 cell differentiation (Figure S9M, Supporting Information).

On the basis that *Plac1* expression is associated with Treg infiltration in the above animal models and that *Plac1* expression is related to poor outcomes in human ICI cohorts in the previous analysis, we hypothesized that *Plac1* is a key that affects the efficacy of immunotherapy. Here we selected the anti‐PD1 therapy, the most common ICI agent used in clinical practice, to establish an immunotherapy model.^[^ ^48^ ^]^ We injected Plac1‐NC and Plac1‐OE cells orthotopically into immune‐competent mice (C3He/J) and administrated anti‐PD1 therapy intraperitoneally three times (IgG served as a negative control) (Figure 5J). Consistent with the subcutaneous tumor model, tumors grew rapidly in the Plac1‐OE group and the weights of mice in this group decreased more dramatically than those of Plac1‐NC group (*p* = 0.0074). The administration of anti‐PD1 could alleviate Plac1‐NC‐induced tumor growth (*p* = 0.0011). while mice in the Plac1‐OE group had the worst OS (*p* = 0.0150) and anti‐PD1 therapy exhibited poor efficacy in them (*p* > 0.05) (Figure 5K–L, Figure S9N, Supporting Information). The above results highlighted the immune regulatory potential of *Plac1* expression whose mechanism required further investigation.

### 2.6 *Plac1* ^+^ Tumor Cells Shape the Immunosuppressive TME Through Recruiting CD4^+^ T Cells and Promoting Treg Differentiation

**Figure 6:**
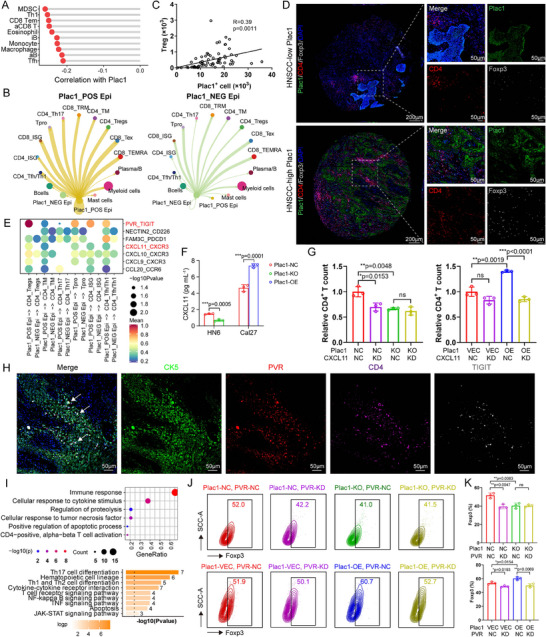
*Plac1^+^* tumor cells shape the immunosuppressive TME through recruiting CD4^+^ T cells and promoting Treg differentiation. A) Lollipop plot shows correlation of different immune cells with *Plac1* expression in the TCGA‐HNSC cohort. B) Comparison of cell–cell interaction strengths of *Plac1^+^* and *Plac1^−^* malignant epithelial cells with other immune cells. C,D) Pearson correlation of *Plac1* ^+^ cell count with Treg count in HNSCC tumor tissues. Representative mIF images of Plac1‐low and ‐high samples are shown in (D) (n = 51). Dapi (blue), Plac1 (green), CD4 (red), Foxp3 (grey), in individual and merged channels are shown. Scale bar, 200 µm and 50 µm. E) Bubble plot shows the interaction pairs between *Plac1* ^+^ and *Plac1* ^−^ malignant epithelial cells and CD4 T subpopulations. F) Quantification of CXCL11 concentration in supernatant of Plac1‐NC, Plac1‐KO, Plac1‐VEC, and Plac1‐OE HNSCC cells (n = 3). G) Quantification of migrated CD4^+^ T cells after cocultured with HNSCC cells of different groups (n = 3). H) Representative mIF images show cell–cell interaction of malignant epithelial cells and CD4^+^ T cells with PVR‐TIGIT pairs. Green: CK5, red: PVR, purple: CD4, grey: TIGIT, blue: Dapi. Scale bar = 50 µm. (I) Representative GO (up) and KEGG pathways (down) enrichment of the predicted target genes expressed in CD4^+^ T cells by Nichenet algorithm. J,K) Representative flow cytometry images (J) and quantification results (K) of Treg ratio of CD4^+^ T cells after coculture with Plac1‐NC/KD (up) and Plac1‐VEC/OE (down) HNSCC cells with different treatments (n = 3). *P* values were calculated by two‐sided Student's *t*‐test in F, G, K, by empirical shuffling in E, and by hypergeometric test in I. *p < 0.05, **p < 0.01, ***p < 0.001.

To determine how *Plac1^+^* epithelial cells shape the immunosuppressive TME and thus promote HNSCC progression, we utilized public bulk RNA‐seq data (TCGA‐HNSC) and in‐house scRNA‐seq data to examine the correlations between *Plac1* expression and other immune cells. First, in the TCGA‐HNSC cohort, *Plac1* expression was strongly negatively correlated with various immune cells, including myeloid‐derived suppressive cells (MDSC) and Th1 cells (**Figure** **6**A). Using the Cellchat algorithm, we quantified the interaction weight of *Plac1* ^+/−^ epithelial cells with other immune cells and found that the differences in interaction weights between groups were more obvious in the CD4^+^ T‐cell subclusters than in other subclusters, which suggested that tumor cell‐CD4^+^ T subcluster interactions are closely associated with *Plac1* expression (Figure 6B, Figure S10A,B, Supporting Information). To validate the correlation of *Plac1* ^+^ tumor cells with CD4^+^ T cells, we performed an IHC assay in the in‐house TMA cohort. Although no significant correlation was observed between the Plac1 IHC score and the CD8^+^ T cell ratio (CD8^+^), macrophage ratio (CD68^+^), or DC ratio (CD11c^+^), the Plac1 expression level was positively correlated with the Treg (Foxp3^+^) ratio (Figure S10C–H, Supporting Information). To further confirm the spatial localization of *Plac1^+^* tumor cells and Tregs, an mIF assay was conducted and *Plac1* ^+^ cells were observed to colocalize with Tregs. Moreover, samples with high Plac1 expression were infiltrated with more Tregs, the numbers of which were positively correlated with each other (R = 0.39, *p* = 0.0011) (Figure 6C‐D). ST directly obtains transcriptomic expression profiles in tissue sections while preserving the spatial location information of each cell in the section, which could help us understand cell‐cell colocalization and interaction in a more direct manner.^[^ ^14^ ^]^ Here, we examined an ST sample from GSE208253^[^ ^49^ ^]^ (Figure S11A, Supporting Information). Spots were identified as epithelial cells by Epcam expression and were divided into *Plac1* ^+^/^−^ epithelial cells according to each cell's *Plac1* expression status. The transcriptomic patterns of six spots adjacent to *Plac1* ^+/−^ epithelial cells (defined as *Plac1* ^+/−^ epi around the region) were subsequently analyzed for “CD4_Tregs”, “CD8_T”, “Macrophage”, and “DC” signature expression. The expression levels of these signatures were compared between *Plac1* ^+^ and *Plac1* ^−^ epi around regions, and consistent with the IHC results, no significant differences were observed in the CD8_T, Macrophage, or DC signatures, whereas CD4_Treg expression was higher in *Plac1* ^+^ epi around region (Figure S11B–E, Supporting Information).

Various ligand‐receptor interactions play crucial roles in immune cell infiltration and direct cell‐cell interactions. Using the ligand‐receptor analysis algorithm CellPhoneDB, we performed ligand‐receptor interaction analysis among *Plac1* ^+^, *Plac1* ^−^ malignant cells, and CD4^+^ T cell subclusters (Figure 6E). Among the secreted factors, CXCL9/CXCR3, CXCL10/CXCR3, CXCL11/CXCR3, and CCL20/CCR6 were more strongly expressed in *Plac1* ^+^ epi‐CD4^+^ T than *Plac1* ^−^ epi‐CD4^+^ T interactions. CXCL11 was found to be restrictedly expressed in *Plac1* ^+^ epithelial cells and its secretion could be induced by *Plac1* expression (*p* = 0.0001) and inhibited by *Plac1* ablation (*p* = 0.0005) (Figure 6F, Figure S12A, C, Supporting Information). We then performed a CD4^+^ T cell Transwell migration assay as previously reported to validate the specific effects of CXCL11 in recruiting CD4^+^ T cells.^[^ ^50^ ^]^ We found that *Plac1* deletion reduced migrated CD4^+^ T cell counts (*p* = 0.0048) and that overexpression of *Plac1* enhanced CD4^+^ T cell migration (*p* = 0.0019). When CXCL11 expression was knocked down by siRNA, both Plac1‐NC HN6 cells (*p* = 0.0153) and Plac1‐OE Cal27 cells (*p* < 0.0001) recruited less CD4^+^ T cells than did the control cells, while no significant differences were seen in Plac1‐KO HN6 cells or Plac1‐VEC Cal27 cells, which suggested that the CD4^+^ T cell recruitment efficacy was associated with the *Plac1* expression level in tumor cells (Figure 6G). The chemoattracting effects of CXCL11 could be further validated when recombinant human (rh)CXCL11 was directly added into the lower chamber (*p* = 0.0256) (Figure S12D, Supporting Information).

After CD4^+^ T cells infiltrate the TME, they interact with *Plac1^+^* tumor cells via direct cell‐cell communication. As displayed in Figure 6E, PVR/TIGIT, NECTIN2/CD226, and FAM3C/PDCD1 were relatively more strongly expressed in *Plac1* ^+^ epi‐CD4^+^ T cells than *Plac1* ^−^ epi‐CD4^+^ T cells and PVR was specific for *Plac1^+^* tumor cells (Figure S12A,B, Supporting Information), which was subsequently validated in HNSCC cells in vitro and in the TMA cohort by mIF (Figure S13A,B, Supporting Information, Figure 6H). To explore the downstream targets of the *Plac1^+^* epi‐CD4^+^ T cell interaction, we performed Nichenet analysis^[^ ^51^ ^]^ and found that pathways related to the immune response and Th cell differentiation were activated in CD4^+^ T cells (Figure 6I, Figure S13C, Supporting Information). Since TIGIT is a canonical inhibitory immune checkpoint that undermines CD4^+^ T cell immune capability,^[^ ^52^ ^]^ we hypothesized that the PVR/TIGIT interaction could induce the differentiation of CD4^+^ T cell into Tregs. To validate our hypothesis, we utilized a tumor cell‐CD4^+^ T cell coculture model. We found that *Plac1* ablation induced a lower Foxp3 ratio (*p* = 0.0083), whereas *Plac1* expression induced an increase in the Foxp3^+^ cell ratio (*p* = 0.0154) and in *Plac1* ^+^ tumor cells, PVR knockdown using siRNA induced a decrease in the Foxp3^+^ cell ratio (Plac1‐NC: *p* = 0.0047, Plac1‐VEC: *p* = 0.0193, Plac1‐OE: *p* = 0.0069) (Figure 6J,K). In addition, we also conducted coculture assays of tumor cells with CD8^+^ T cells and macrophages according to a previous report to evaluate whether *Plac1* expression influences the phenotypes of CD8^+^ T cells and macrophages.^[^ ^53^ ^]^ However, no significant differences were observed in the expression of CD8^+^ T cell exhaustion markers or immunosuppressive marker expression in macrophages between groups, which indicated that the *Plac1‐*associated immune regulatory effects were specific to CD4^+^ T cells (Figure S13D–G, Supporting Information). Overall, we delineated cell–cell interaction landscape of tumor cells and immune cells and revealed that *Plac1* ^+^ tumor cells recruited CD4^+^ T cells through the CXCL11/CXCR3 axis and then induced Treg differentiation through PVR/TIGIT to shape the immunosuppressive TME of HNSCC.

### 2.7 Tregs Enhance the Malignant Biological Characteristics of *Plac1* ^+^ Cells

**Figure 7:**
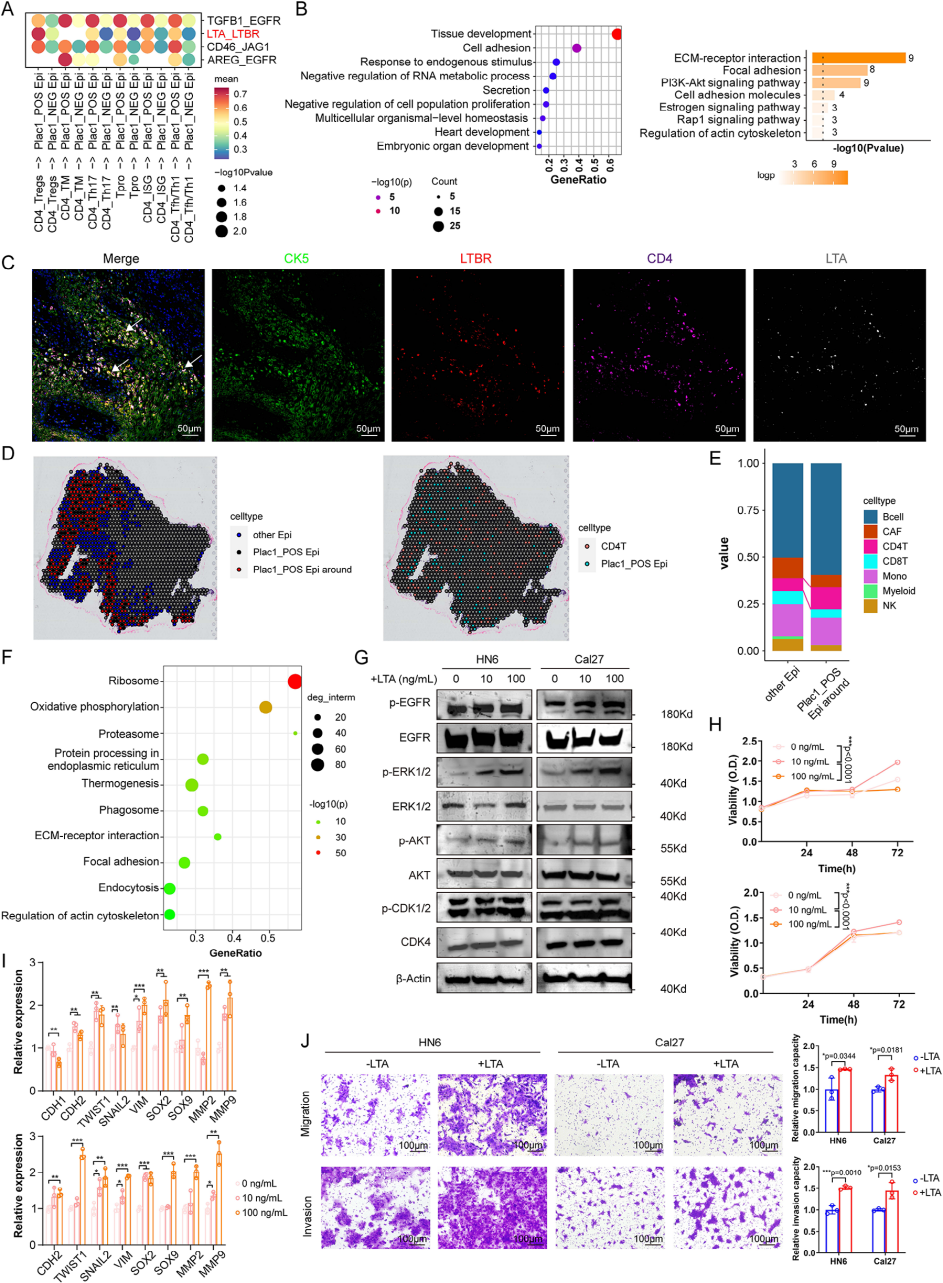
Tregs enhance the malignant biological characteristics of *Plac1* ^+^ cells. A) Bubble plot shows the interaction pairs between CD4 T subpopulations and *Plac1* ^+^ and *Plac1* ^−^ malignant epithelial cells. B) Representative GO (left) and KEGG pathways (right) enrichment of the predicted target genes expressed in *Plac1* ^+^ malignant epithelial cells. C) Representative mIF images show cell‐cell interaction of CD4^+^ T cells and malignant epithelial cells with LTA‐LTBR pairs. Green: CK5, red: LTBR, purple: CD4, grey: LTA, blue: Dapi. Scale bar = 50 µm. D,E) Infiltration pattern (D) and quantification (E) of CD4^+^ T cells in dots around *Plac1* ^+^ malignant and other epithelial cells. (F) KEGG enrichment analysis of genes expressed in dots positive with PVR/TIGIT, related to (D) and (E). G) WB analysis of the levels of PI3K/AKT pathway proteins of HNSCC cells treated with LTA in different concentrations. H) Cell viability of HN6 (up) and Cal27 (down) cells treated with LTA in different concentrations (n = 3). I) mRNA expression level of EMT‐related genes of HN6 (up) and Cal27 (down) cells treated with LTA in different concentrations (n = 3). J) Representative images (left) and quantification (right) of Transwell assay of HNSCC cells treated with or without LTA (n = 3). *P* values were calculated by two‐sided Student's *t*‐test in H, I, J, by empirical shuffling in A, and by hypergeometric test in B, F. *p < 0.05, **p < 0.01, ***p < 0.001.

Finally, considering the colocalization of Tregs and *Plac1* ^+^ tumor cells, we wondered whether Tregs in turn affect malignant cells and which cell‐cell interaction molecules induce these effects. Therefore, we again used the CellphoneDB algorithm and set CD4^+^ T subpopulations as senders and *Plac1* ^+/−^ cells as receivers. We found that TGFB1/EGFR, LTA/LTBR, CD46/JAG1, and AREG/EGFR ligand‐receptor pairs were stronger in CD4^+^ T cell‐*Plac1* ^+^ tumor cells than in CD4^+^ T cell‐*Plac1* ^−^ tumor cells, among which LTA/LTBR expression was highly specific for Tregs and *Plac1* ^+^ cells (**Figures** **7**A, S14A,B, Supporting Information). Nichenet analysis revealed that Tregs interact with *Plac1* ^+^ cells and activate downstream genes associated with malignant characteristics, such as “Cell adhesion”, “ECM‐receptor interaction”, and “PI3K‐AKT signaling pathway” (Figure 7B, Figure S14C, Supporting Information). The ligand‐receptor pair LTA/LTBR was validated by mIF assay in our validation TMA cohort (Figure 7C). We also analyzed ST samples for validation and a higher ratio of CD4^+^ T cell infiltration around *Plac1* ^+^ epi was observed (Figure 7D,E). The genes expressed in these dots were enriched in pathways associated with “Phagosome”, “ECM‐receptor interaction”, and “Regulation of actin cytoskeleton”, which was consistent with the results of Nichenet analysis and further supported our hypothesis (Figure 7F). Next, we validated these malignancy‐inducing effects in vitro by adding rhLTA (Origene, #TP303741) into the medium of HNSCC cells. WB analysis of PI3K/AKT pathway proteins revealed that, upon treated with rhLTA, PI3K/AKT pathway‐related proteins were activated in HN6 and Cal27 cells in a concentration‐dependant manner (Figure 7G). Since the phosphorylation level of cell‐cycle checkpoint CDK1/2 was increased, we evaluated the viability of HNSCC cells treated with different rhLTA concentrations and observed significantly higher cell viability (Figure 7H). Since the downstream genes were also enriched in “ECM‐receptor interaction” and “Focal adhesion” (Figure 7B), which were associated with epithelial‐mesenchymal transition (EMT), cell migration, and invasion characteristics, we evaluated EMT‐related genes via qPCR and migration and invasion ability via Transwell assay. As expected, stimulation with rhLTA led to the upregulation of EMT‐related genes and increased HNSCC cell migration and invasion ability (Figure 7I–J). In conclusion, in the *Plac1* ^+^ tumor cell‐Treg interaction, Tregs could enhance the malignant characteristics of *Plac1^+^* tumor cells through the LTA/LTBR axis and by PI3K/AKT pathway activation.

## 3 Discussion

As a critical factor in embryogenesis, germline tissue development, and functional maintenance, CTA expression is strictly restricted and finely regulated.^[^ ^54^ ^]^ Aberrant expression of CTAs may lead to dysfunction of gamete and the reproductive system, and as recently reported, tumorigenesis and tumor progression. The association of CTA reactivation with tumorigenic signaling has been reported in solid tumors, including colorectal cancer, liver cancer, and breast cancer.^[^ ^5^, ^6^, ^55^ ^]^ However, in the context of HNSCC, whether CTA genes contribute to HNSCC initiation and progression and how they affect the TME remain unclear. In the present study, we identified a specific CTA gene expressed in HNSCC tumor cells, *Plac1*. We then identified a subpopulation of tumor cells that expresses *Plac1* and termed them “*Plac1^+^* tumor cells”. Notably, *Plac1* ^+^ tumor cells not only enhance tumor malignant characteristics and tumor growth in a cancer‐autonomous way, but also shape an immunosuppressive TME characterized by Treg infiltration. Our study revealed that *Plac1* functions as a key tumor‐promoting and immunoregulatory CTA gene in HNSCC.

*Plac1* was first reported to be widely expressed during fetal development, and its expression level is downregulated during embryonic maturation.^[^ ^56^ ^]^ Interestingly, its expression is reactivated in HNSCC. In the present study, we screened candidate regulators of *Plac1* from embryogenesis and HNSCC scRNA‐seq data. Notably, we found that *SP1* could induce *Plac1* expression during HNSCC initiation and progression, which was validated by the CUT&Tag‐seq assay and was consistent with previous reports.^[^ ^57^ ^]^ Our findings lay the foundation for further investigations into the mechanism of carcinogenesis and the development of therapeutics for HNSCC.

Our previous work revealed that *Plac1* expression was associated with immunosuppressive TME and could be a prognostic indicator of poor outcomes in ICI cohorts.^[^ ^24^ ^]^ Importantly, in this study, we did not only reveal how *Plac1* ^+^ tumor cells induce Treg‐related immunosuppressive TME, but also discovered an interaction loop between *Plac1* ^+^ tumor cells and Tregs. After CD4^+^ T cells are recruited into the TME, the highly expressed ligand PVR on *Plac1^+^* tumor cells binds to the canonical immune checkpoint TIGIT on CD4^+^ T cells and induces their differentiation into Tregs.^[^ ^58^ ^]^ Crucially, the inhibition of PVR on tumor cells significantly disrupts Treg transformation of CD4^+^ T cells. Moreover, although the effects of Tregs on other immune cells, such as the inhibition of cytotoxic T cell functions, have been previously reported,^[^ ^59^ ^]^ few studies have revealed how Tregs affect tumor cells. In the present study, we found that Tregs in the TME of HNSCC could secrete LTA, interacting with its receptor LTBR on *Plac1^+^* tumor cells and activating the downstream PI3K/AKT signaling pathway to promote HNSCC cell proliferation, migration, and invasion. This reciprocal interaction and positive feedback loop represent promising targets for immunotherapy in HNSCC.

Several limitations of this study should be acknowledged. First, the molecular mechanism of the protumor effects of *Plac1* requires further investigation. For example, how *Plac1* expression enhances caveolae‐related gene upregulation, and how *Plac1^+^* tumor cells secrete higher concentrations of CXCL11 and express higher levels of PVR. Second, the animal tumor models constructed in this study cannot fully simulate HNSCC in human patients. Preclinical models, such as patient‐derived organoid (PDO) and patient‐derived xenograft (PDX) models, should be used to assess whether our findings are still valid in the context of human tumors. Furthermore, the potential of *Plac1* as a therapeutics target has not been investigated. We have demonstrated that *Plac1* plays an important role in promoting HNSCC progression and shaping the immunosuppressive TME and we hypothesized that *Plac1* could be a promising molecular target for HNSCC treatment, which will be pursued in our future investigation.

## 4 Conclusion

**Figure 8:**
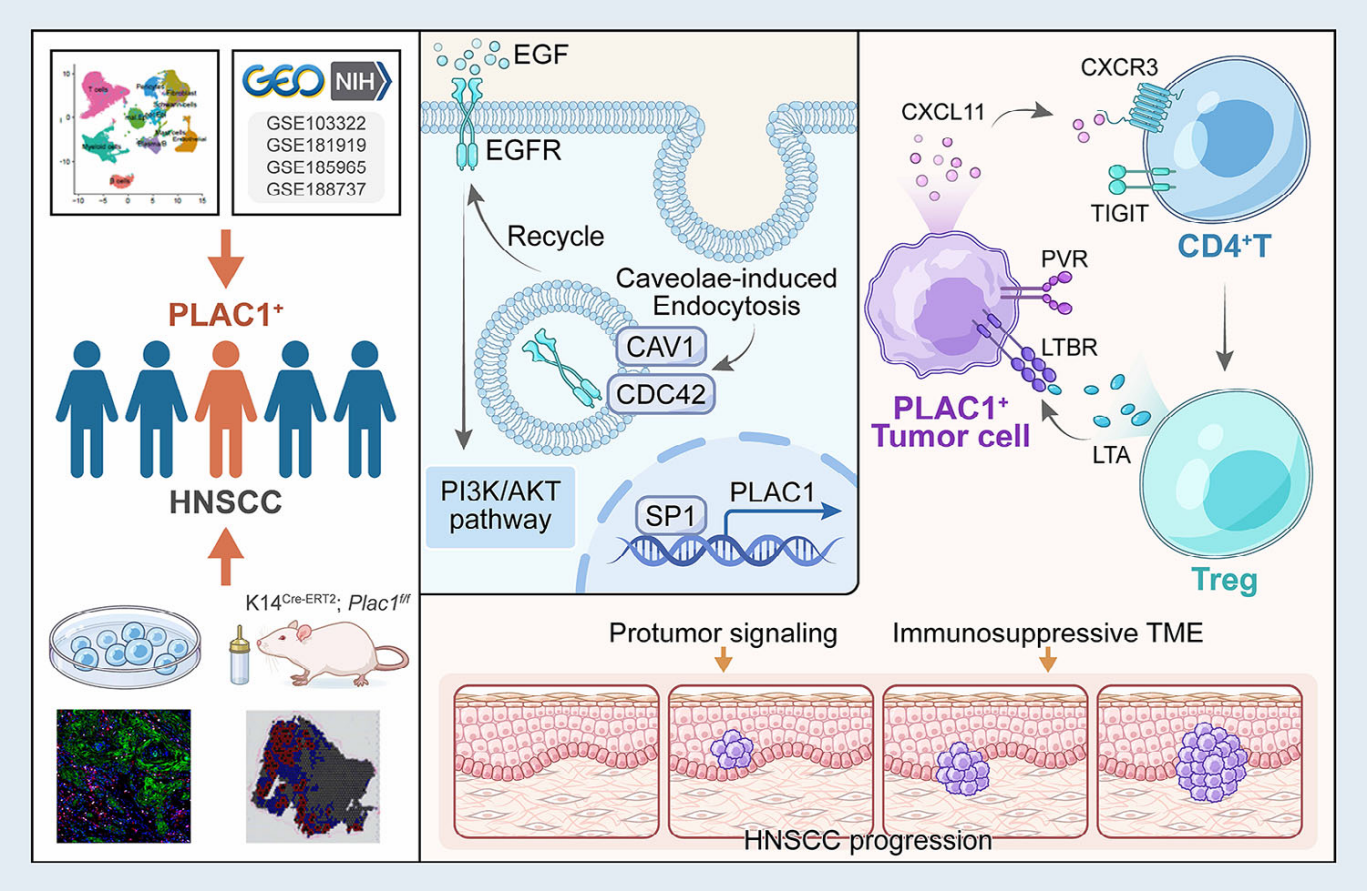
Schematic illustration of tumor‐promoting and immunosuppressive effects of the HNSCC‐specific CTA, *Plac1*.

In conclusion, our findings demonstrate that one of the CTA genes, *Plac1*, is specifically expressed in HNSCC tumor cells and this gene promotes tumor progression through a dual mechanism involving both the enhancement of PI3K/AKT pathway and the induction of Treg differentiation. These findings suggest that *Plac1* is a promising target for immunotherapy in the HNSCC context (**Figure** **8**).

## 5 Experimental Section

### Patient Information and Tissue Specimens

HNSCC diagnosis was based on histopathological features, including depth of invasion, pathological stage, and lymph node metastasis. Tumor tissues and paired adjacent normal tissues from three patients with HNSCC were collected and frozen immediately in liquid nitrogen within 30 min after surgery. Analyses were performed on these samples using bulk RNA‐seq. The in‐house scRNA‐seq cohort and TMA validation cohort were described in our previous study.^[^ ^13^ ^]^ This study was reviewed and approved by the local medical ethics committee of Shanghai Ninth People's Hospital, Shanghai Jiao Tong University School of Medicine. Written informed consent was obtained from each patient prior to sample collection. All experimental methods abided by the Helsinki Declaration.

### Cell Lines and Cell Cultures

HNSCC cell lines Cal27, HN6, SCC7 cells, and monocyte cell line THP‐1 were obtained from FuHeng Biology Company (Shanghai, China) as described previously.^[^ ^60^ ^]^ Human CD4^+^ T cells and CD8^+^ T cells were isolated from the peripheral blood of healthy donors by using the EasySep Human CD4^+^ T‐Cell Isolation Kit (Stemcell, #17 952) and EasySep Human CD8^+^ T‐Cell Isolation Kit (Stemcell, #17 953). Cal27 and HN6 cells were cultured in Dulbecco's Modified Eagle Medium (DMEM) (GIBCO, #C11995500BT) supplemented with 10% fetal bovine serum (FBS) and 1% penicillin‐streptomycin. SCC7, THP‐1, and T cells were cultured in Roswell Park Memorial Institute (RPMI) 1640 medium (GIBCO, #C11875500BT) supplemented with 10% FBS. ImmunoCult™ Human CD3/CD28 T Cell Activator (Stemcell, #10 971) was added into T cell medium (25 µL mL^−1^) for cell activation, and human recombinant IL‐2 (Stemcell, #78 036) was added into T cell medium (50 IU mL^−1^) for cell expansion. To induce macrophage differentiation, cells were seeded at a density of 10^6^ cells in a 6 well plate in the presence of 100 ng mL^−1^ PMA (MCE, #HY‐18739) for 48 h.^[^ ^61^ ^]^

### Mice

All animal experiments were approved by the Animal Care and Use Committee of Shanghai Ninth People's Hospital, Shanghai Jiao Tong University School of Medicine, Shanghai, China. Plac1^f^ *^/^* ^f^ mice were provided by Cyagen Biosciences Inc (Suzhou, China), and K14^Cre‐ERT2^ mice were purchased from The Jackson Laboratory, both of which were backcrossed to C57BL/6J backgrounds. C3H/HeJ mice were purchased from Beijing Vital River Laboratory Animal Technology Co., Ltd. (Beijing, China) and Balb/c nude mice were purchased from Shanghai Jihui Laboratory Animal Care Co., Ltd. (Shanghai, China). All mice were maintained under pathogen‐free conditions in the animal care facilities of Shanghai Ninth People's Hospital, Shanghai Jiao Tong University School of Medicine. All animals were maintained at room temperature and with free access to food and water with a 12‐h light/dark cycle. All mice used in experiments throughout the study exhibited normal health. All mice were used after 1 week of acclimatization to the facility. All animal studies were performed in accordance with the Guide for Care and Use of Laboratory Animals (The Ministry of Science and Technology of China, 2006).

### RNA‐Seq and Data Analysis

Total RNA extraction was performed using a TRIzol reagent according to the manufacturer's protocol (Takara). MRNA library construction was performed on an Illumina HiSeq X Ten platform (Sinotech genomics Co., Ltd, Shanghai, China). Raw reads in fastq format for each sample were generated and processed using Trimmomatic.^[^ ^62^ ^]^ Clean reads for each^[^ ^63^ ^]^ sample were retained by removing low‐quality reads and mapped to the genome using HISAT2 for subsequent analyses. RSEM was used to quantify gene expression levels (FPKM). The NOISeq method was used to screen out DEGs between two groups with a fold change ≥ 2 and divergence probability ≥ 0.8.

### CUT&Tag‐Seq and Data Analysis

HN6 cells were harvested, counted, and centrifuged at 300 × g for 5 min at room temperature. A total of 100 000 cells/samples were subjected to CUT‐Tag according to the manufacturer's instructions (Vazyme, #TD904). Two duplicates of HN6 cells were set. Anti‐SP1 (Abcam, #Ab231778) and anti‐IgG negative control antibodies (CST, #2729) were used. After sample preparation, CUT&Tag‐seq experiments were performed by Sinotech genomics Co., Ltd (Shanghai, China). The size distribution of libraries was determined by Agilent 4200 TapeStation analysis and libraries were mixed to achieve equal representation as desired aiming for a final concentration as recommended by the manufacturer. Paired‐end Illumina sequencing was performed on the barcoded libraries following the manufacturer's instructions. Paired‐end reads were aligned using Bowtie2 version 2.2.5 with options: ‐local‐very‐sensitive‐local‐no‐unal‐no‐mixed‐no‐discordant‐phred33 ‐I 10 ‐X 700. For peak calling, the parameters used were macs2 call peak ‐t input ‐c igG ‐q 0.05 ‐f BEDPE/BED ‐keep‐dup all ‐n out name. To visualize the genomic occupancy of the peaks ± the 3 kb flanking TSSs, BAM files were converted to BIGWIG files with the bamCoverage function of deep‐Tools^[^ ^64^ ^]^ with the parameter “–normalizeUsing RPKM” and “computeMatrix”, “*plotHeatmap*” functions were applied. For genome browser representation, the BIGWIG files were loaded using the Integrative Genomics Viewer (IGV).^[^ ^65^ ^]^

### Phosphoproteomics Study and Bioinformatics Analysis

The cell pellets were lysed by 8 mol L^−1^ urea in 100 mmol L^−1^ Tris‐HCl (pH 8.5) with a protease cocktail to denature proteins and subjected to two rounds of sonication on ice to disrupt protein‐protein and DNA‐protein interactions. The protein concentration was measured, and equal amounts of protein were used for tryptic digestion. TCEP (Thermo Scientific) and iodoacetamide (final concentration is 10 mmol L^−1^) (Sigma) for reduction and alkylation, respectively, were added to the solution and incubated at room temperature for 30 min. The protein mixture was diluted fourfold and digested with trypsin at a 1:50 ratio (w/w) (Promega) at 37 °C overnight. The tryptic‐digested peptide solutions were desalted on MonoSpin C18 columns (GL Science), and the eluents were dried in a speed vacuum. The peptide mixtures were finally dissolved in HEPES buffer (100 mmol L^−1^, pH 8.0). The peptide concentrations were measured with a Quantitative Colorimetric Peptide Assay (Thermo Scientific) before TMT labeling to ensure that the number of peptides in each channel for TMT labeling was equal. TMT6plex amino‐reactive reagents (0.8 mg per vial, Thermo Scientific) were suspended in 41 µL of anhydrous acetonitrile and were suitable for labeling the 100 µg peptide solutions in each channel. Reactions were allowed to proceed at room temperature for 1 h and then quenched by the addition of 8 µL of 5% hydroxylamine for 15 min. The TMT‐labeled samples were pooled at a 1:1:1:1:1:1 ratio. The mixture was vacuum centrifuged to near dryness and desalted on a MonoSpin C18 column (GL Science). The desalted TMT‐labeled peptides were subjected to offline prefractionation with a Thermo Fisher Pierce high pH reversed‐phase separation kit (#84 868) according to the manufacturer's user guide. We separated the complex peptide mixture into 8–10 fractions. All the fractions for each TMT experiment were analyzed by separation on a homemade 30 cm‐long pulled‐tip analytical column (75 µm ID x 360 µm OD, ReproSil‐Pur C18‐AQ 1.9 µm resin, Dr. Maisch GmbH). The column was then placed in‐line with an Easy‐nLC 1200 nano HPLC instrument (Thermo Scientific) for mass spectrometry analysis. The analytical column temperature was set at 55 °C during the experiments. The mobile phase and elution gradient used for peptide separation were as follows: 0.1% formic acid in water as buffer A and 0.1% formic acid in 80% acetonitrile as buffer B; 0–1 min, 5%‐8% B; 1–104 min, 8%‐35% B; 104–114 min, 35%–50% B, 114–115 min, 50%‐100% B, 115–120 min, 100% B. The flow rate was set at 300 nL min^−1^. Data‐dependent tandem mass spectrometry (MS/MS) analysis was performed with an Orbitrap Eclipse Tribrid mass spectrometer (Thermo Scientific). Peptides eluted from the LC column were directly electrosprayed into the mass spectrometer with a distal 2.1‐kV spray voltage. A 3 s cycle of one full‐scan MS spectrum (m/z 400–1 600) was acquired, followed by acquisition in top speed mode (first mass fixed at a 100 m/z scan range) at a 38% normalized collision energy. The full scan resolution was set to 120 000 with an automated gain control (AGC) target of 4e5. The MS/MS scan resolution was set to 50 000 with an isolation window of 1.2 m/z and an AGC target of 1.25e5. The number of microscans was one for both the MS and MS/MS scans, and the maximum ion injection times for the MS and MS/ MS scans were 50 and 100 ms, respectively. The dynamic exclusion settings used were as follows: charge exclusion, 1 and > 8; exclude isotopes, on; and exclusion duration, 60 s. The acquired MS/MS data were analyzed against a UniProtKB human database (released on Sept. 30, 2018) by MaxQuant V1.6.10.43 using the default setting. The tolerance of the precursor mass and fragment mass were set to ±20 ×10^−6^. The main search peptide tolerance was set at 4.5 ×10^−6^ according to the features of the instrument in the study. Carbamidomethylation of cysteine +57.021 Da) and acetylation of the protein N‐terminus were set as static modifications. The quantification search type selected was reporter ion ms2 using the TMT 10plex method. Oxidation of methionine (+ 15.995 Da) was set as a variable modification. Trypsin was defined as the cleavage enzyme, and the maximum number of missed cleavages was set at 2. All identified proteins had an FDR ≤ 1%, which was calculated at the peptide level. The unique peptides were selected for protein quantitation.

The interpretation of the proteomic results from MaxQuant was performed with the R4.0.0 platform. Briefly, P was adjusted by the Benjamini and Hochberg method using the limma package built for the R environment. Proteins were required to have a |log_2_ (fold change)| ≥ 0.26 and an adj.P value of 0.05 to be considered differentially expressed. GO analysis was performed using the DAVID tool (https://david.ncifcrf.gov/).

### Dimension Reduction and Clustering Analysis of scRNA‐seq Data

Cell clustering was performed after data normalization. The effects of the ribosomal gene percent and mitochondrial gene percent were regressed when scaling gene expression. To remove potential batch effects, we used canonical correlation analysis (CCA).^[^ ^66^ ^]^ The “*RunUMAP*” function implemented in Seurat (v4.0.3)^[^ ^67^ ^]^ was used to reduce dimensionality. Cell clusters were identified using the “*FindClusters*” function in Seurat. To identify marker genes for each subcluster, the expression profile of a subcluster was contrasted with those of the other subclusters using the Seurat “*FindAllMarkers*” function. DEGs were defined as Bonferroin‐corrected P‐value ≤ 0.05 and an average |log_2_ (fold change)| ≥ 0.25. We annotated the clusters as different cell subtypes based on their average gene expression of well‐known markers or specifically expressed marker genes.

Copy number instability was assessed with the R package inferCNV (v1.8.19),^[^ ^68^ ^]^ which is designed to infer copy number alterations from tumor scRNA‐seq data. This package compares the expression intensities of genes across epithelial cells with non‐epithelial cells (e.g., T cells, B cells, and endothelial cells). By this means, we identified malignant and non‐malignant cells.

### Screening Strategy of HNSCC‐Related CTA

To find out which CTA was associated with HNSCC tumor lesions, we screened CTA by their expression patterns in both bulk RNA‐seq data and scRNA‐seq data of HNSCC. As for bulk RNA‐seq data, the eligible CTA should be highly expressed in tumor tissues rather than adjacent normal tissues; as for scRNA‐seq data, the eligible CTA should be highly expressed in malignant epithelial cells rather than normal epithelial cells. Then we overlapped genes in different datasets and obtained *Plac1* as our candidate for further investigation.

### Trajectory Analysis of Embryo Epithelial Cells

To clarify the differentiation trajectory of epithelial cells during embryogenesis, Monocle (v2.24.0)^[^ ^69^ ^]^ was used to illustrate the differentiation of epithelial cells from public datasets. First, the count matrices and metadata information to create a new CellDataSet object. The count matrices had been normalized as log_2_TPM. Then, the following setting was used to infer single‐cell trajectory: expressionFamily = gaussianff. During the construction of the single‐cell trajectories, the *VariableFeatures* function in Seurat (v4.0.3) was first used to filter a list of gene IDs to be used for defining progression. Then, dimensional reduction was performed using the ICA method. Finally, cells of 2‐cell were set as the root. The results were visualized using the function *plot_cell_trajectory*, with color_by = “DefineTypes”, “Pseudotime” or “State”.Gene expression levels of *Plac1*, *SP1*, and *OTX2* were also calculated along with the pseudotime axis.

### Analysis of Plac1 Upregulation‐Related TFs

To analyze TFs that could potentially regulate *Plac1* expression in HNSCC, potential TFs of *Plac1* were retrieved from four TF‐related public datasets: GTRD, htftarget, JASPAR87, and chIPBase. The TF genes from four datasets were overlapped and resulted in 2 candidate TFs for *Plac1*. Then their expression trends along embryogenesis pseudotime axis were evaluated and their expression correlation with *Plac1* expression in the in‐house scRNA‐seq dataset, GSE103322, and GSE181919 were calculated.

### Functional Enrichment Analysis

Functional annotation of a list of genes of interest was performed with the KEGG database and GO classification database. Enrichment analysis of GO categories was performed with the R clusterProfiler (v3.14.3) package, and pathway enrichment analysis was tested upon verification of a hypergeometric distribution by R “*phyper*” function. Within the enriched GO categories, similar categories containing a large percentage of overlapping genes were summarized into one category.

### Survival Analysis of ICI Cohorts

Gene expression information collected from public ICI datasets (GSE91061,^[^ ^26^ ^]^ Gide2019_PD1 cohort,^[^ ^25^ ^]^ Nathanson2017 cohort,^[^ ^27^ ^]^ and IMvigor210 cohort^[^ ^28^ ^]^). The “*surv_cutpoint*” function of R package survminer, an outcome‐oriented method providing a value of a cut‐point that corresponds to the most significant relation with outcomes, was used to perform dichotomy of *Plac1* expression and to divide the patients into two groups according to the selected maximum logarithm statistics. The two‐sided long‐rank test was used to compare Kaplan–Meier survival curves. The comparison of *Plac1* expression between R and NR patients was determined by two‐sided Student's *t* tests.

### Estimation of Correlation of Immune Infiltration Levels with Plac1 Expression

To evaluate the correlation of immune infiltration levels with *Plac1* expression, gene signatures of 28 tumor‐infiltrating lymphocytes (TILs) were first obtained from the TISIDB database (http://cis.hku.hk/TISIDB).^[^ ^70^ ^]^ Subsequently, the ssGSEA algorithm was employed from the GSVA R package^[^ ^71^ ^]^ to estimate the immune cell enrichment scores for each tumor sample. The correlations of immune scores with *Plac1* expression were calculated by Pearson's correlation test.

### Cell–Cell Communication Analysis

CellChat (v1.6.1) was used to infer cell–cell communication by integrating scRNA‐seq data with the ligand‐receptor interaction database CellChatDBHuman.^[^ ^72^ ^]^ CellChat quantifies the communication probability between two interacting cell groups based on the average expression values of a ligand and a receptor as the cofactors. The calculated communication probabilities are assigned as edge weights to quantify the interaction strength. Additionally, CellChat computes the communication probability at the signaling pathway level by summarizing the communication probabilities of all ligand‐receptor interactions associated with each signaling pathway. To identify signaling changes, the differential outgoing and incoming interaction strengths of this cell population in each cell‐cell communication network between two conditions were calculated and compared.

The R package NicheNetR (v2.0.6)^[^ ^51^ ^]^ was used to infer mechanisms of interaction in *Plac1* ^+/^ *^−^* tumor cells and CD4^+^ T cell subpopulations. For ligand and receptor interactions, clustered cells with gene expression over 10% were considered. The top 100 ligands and top 1000 targets of DEGs of “sender cells” and “receiver cells” were extracted for paired ligand‐receptor activity analysis. The function *ligand_activity_target_heatmap* in Nichenet_output was used to display the regulatory activity of ligands.

### ST Data Analysis

ST data was downloaded from public datasets. The gene‐spot matrices generated after ST data processing from ST and Visium samples were analyzed with the Seurat package (v4.0.3) in R. Spots were filtered for minimum detected gene count of 200 genes while genes with fewer than ten read counts or expressed in fewer than three spots were removed. Signature scoring derived from scRNA‐seq or ST signatures was performed with the *AddModuleScore* function with default parameters in Seurat. Spatial feature expression plots were generated with the *SpatialFeaturePlot* function in Seurat (v4.0.3).

### Plasmids Transfection

*Plac1* expression plasmid was purchased from Ibsbio Co. Ltd (Shanghai, China). The plasmid was constructed with His‐tag. For transfection, lipofectamine 3000 (Thermo Fisher, #L3000075) and plasmid with a proportion of 2.5:1 was diluted with 250 µL serum‐free Opti‐MEM (Thermo Fisher, #31 985 070) respectively. Following gentle mixing, they were incubated for 20 min and then added into cells at room temperature. After being transfected, the cells were incubated at 37 °C, in 5% CO_2_, for 8–12 h, and the culture medium was then replaced with DMEM added with 10% FBS. If not noted specifically, the cells were used for further experiments or collected after 48 h of transfection.

### siRNAs Transfection

siRNAs specific for different targets were purchased from Shanghai Genepharma Co., Ltd (Shanghai, China). A scrambled nontargeting siRNA was used as negative control. The cells were transfected with 100 nM siRNAs diluted in Opti‐MEM using Lipofectamine 3000 according to manufacturer's protocols. The sequences of siRNAs against different targets were as follows:

Plac1: F’: GCUCCAUAGACUGGUUCAUTT, R’: AUGAACCAGUCUAUGGAGCTT;

SP1: F’: GCCGUUGGCUAUAGCAAAUTT, R’: AUUUGCUAUAGCCAACGGCTT;

CXCL11: F’: CGAUGCCUAAAUCCCAAAUTT, R’: AUUUGGGAUUUAGGCAUCGTT;

PVR: F’: GGGAUCGGGAUUUAUUUCUTT, R’: AGAAAUAAAUCCCGAUCCCTT.

### Generation of Plac1 Genetic Knockout in Human HNSCC Cells by CRISPR/cas9 System

A human *Plac1*–targeting CRISPR plasmid was first constructed against the sequence: GAAGGATGAGAAATGCTACG with vector pCLenti‐U6‐spgRNA v2.0‐CMV‐EGFP‐WPRE. The human *Plac1*–targeting vector or empty vector was transfected into HNSCC cells with Cas9 plasmid and selected by puromycin (3 µg mL^−1^) for 3 days. The transfected cells were split into 96 wells of plates by gradient dilutions to generate single cell–derived individual clones. The genomic alignment was conducted to confirm the mutation and protein expression was measured by WB using anti‐Plac1 antibody (Absin, #Abs103873) to characterize the deletion efficiency.

### Cell Proliferation, Migration, and Invasion Assays

HNSCC cells with genetic interventions or reagent treatments were seeded in 96‐well plates at a concentration of 1000 cells per well and cultured at 37 °C for 3 or 4 days. At the designated detection timepoint, the culture medium in each well was replaced by 100 µL fresh DMEM with 10 µL CCK8 and incubated at 37 °C for 2 h. The absorbance of each well was measured by a microplate reader (Tecan Infinite 200) at a wavelength of 450 nm.

For the migration assay, genetically modified or reagent treated cells were seeded into a Transwell chamber (8 µm, Corning, #3422) in DMEM without FBS and then cultured in 24‐well plates for 24 h. Medium with 10% FBS was added to the lower compartments as a chemoattractant. Then, the migrated cells were fixed with 4% paraformaldehyde for 20 min and treated with 0.1% crystal violet for 1 h. The cells in the bottom compartment of the chamber were counted under a microscope in at least 5 randomly selected fields. For the invasion assay, the top chamber was coated with Matrigel (1:10 in DMEM dilution, Corning), the other procedures were the same as those used for the Transwell migration assay.

### Apoptosis Analysis by Flow Cytometry

HNSCC cells with genetic interventions were seeded in 12‐well plates. After incubation for 24 h, cells were then subjected to flow cytometry for apoptosis analysis by using an Annexin V‐FITC/PI detection kit (BD Biosciences, #556 547). The ratio of early apoptosis and late apoptosis were calculated following the manufacturer's instructions.

### ICC Analysis with Confocal Microscopy

HNSCC cells with genetic alteration were seeded into confocal dishes (NEST, #801 001) with a φ of 20mm. After 24 h, cells were treated with recombinant human epithelial growth factor (rhEGF) (R&D system, #236‐EG‐200) at 50 ng mL^−1^ for 5 min. Then, cells were fixed with 4% paraformaldehyde for 15 min, permeabilized in 0.1% Triton X‐100 or saponin for 10 min, and then blocked with 1% BSA for 30 min. Primary antibodies against EGFR (Abcam, #Ab52894) were added and incubated overnight at 4 °C. After washing three times, the cells were then treated with Alexa Fluor 488‐conjugated secondary antibodies (Jackson, #111‐545‐003) for 1 h at room temperature and subjected to nuclear staining with DAPI (Sigma, #D9542) for 10 min. Finally, images were captured with a Confocal laser scanning microscope (CLSM, Leica SP8).

### Flow Cytometric Analysis of EGFR on HNSCC Cells

To evaluate the abundance of EGFR expression on HNSCC cells, genetically altered HNSCC cells were seeded in 12‐well plates and pre‐treated with CHX (MCE, #HY‐12320) at 20 µM for 2 h and then incubated with rhEGF at 50 ng mL^−1^ for 5 min. The cells were collected, washed, and incubated with anti‐EGFR antibody (Biolegend, #352 906) for 30 min. The cells were subjected to analysis by flow cytometry (BD Biosciences). As for the endocytosis inhibition, Pitstop2 (MCE, #HY‐115604, 20 µM), Filipin III (MCE, #HY‐N6718, 800 nM), and Genistein (MCE, #HY‐14596, 2 µg mL^−1^) were administrated for 2 h before EGF treatment.

### Coculture of Tumor Cells with CD4^+^ T cells and CD8^+^ T cells

HNSCC cells were seeded in 12‐well plates and underwent genetic interventions. After incubation for 24 h, CD4^+^ or CD8^+^ T cells were added to the medium of HNSCC cells at a ratio of 1:1 in triplicate and directly cocultured for another 48 h. Then the T cells were collected for immunophenotyping by flow cytometry (BD Biosciences). Anti‐CD4 (Biolegend, #317 408), anti‐CD8 (Invitrogen, #45‐0088‐41), anti‐Foxp3 (Biolegend, #320 114), anti‐LAG3 (Biolegend, #369 312), anti‐PD1 (Biolegend, #367 412), anti‐TIGIT (Biolegend, #372 704), and anti‐TIM3 (Biolegend, #345 012) were used. As for the in vitro transmigration assay, 5 × 10^6^ CD4^+^ T cells were planted on the top chamber of the 24‐well plate Transwell system (5 µm, Corning, #3421), HNSCC cells with different genetic interventions were pre‐seeded in the lower chamber as a chemoattractant. 24 h later, cells from top chamber migrated towards tumor cells were collected for flow cytometry to enumerate transmigrated cell counts in different groups. To investigate the chemoattract effects of CXCL11, rhCXCL11 (MCE, #HY‐P7229) was added into the medium of the lower chamber at the concentration of 2 ng mL^−1^ and 24 h later, migrated cells were processed as described above.

### Coculture of Tumor cells with THP‐1‐Derived Macrophages

^[^ ^53^ ^]^A Transwell chamber (0.4 µm, Corning, #3412) was utilized for the coculture assay. HNSCC cells with different genetic intervention were seeded in the top insert of the Transwell and cocultured with THP‐1‐derived macrophages at a ratio of 1:2 in triplicate. After 5 days, THP‐1‐derived macrophages were analyzed using RT‐qPCR. The primer information can be found in Table S1 (Supporting Information).

### Enzyme Linked immunosorbent assay (ELISA)

Supernatants from Plac1‐OE, Plac1‐KO, and control cells were analyzed using the human CXCL11 ELISA kit (Multi Sciences, #EK1207) following the manufacturer's instructions.

### WB

Proteins were extracted from cultured cells using cold RIPA lysis buffer (Thermo Fisher, #89 901), and protease inhibitor cocktail (Bimake, #B14001) and a phosphatase inhibitor cocktail (Bimake, #B15001). Proteins from cell lysates were separated by SDS‐PAGE and then transferred to PVDF membranes (Bio‐Rad, #1 620 175). The blots were incubated with primary antibodies at 4 °C overnight and with secondary antibodies at room temperature for 1 h. Blot images were captured by ODYSSEY imaging system. Primary antibodies used in the current investigation were listed below: anti‐β‐actin (CST, #3700), anti‐Plac1 (Absin, #Abs103873), anti‐His tag (Abcam, #Ab18184), anti‐CDC42 (Abcam, #Ab187643), anti‐CAV1 (Abcam, #Ab32577), anti‐pEGFR (CST, #2237), anti‐EGFR (CST, #4267), anti‐pERK1/2 (Abcam, #Ab201015), anti‐ERK1/2 (CST, #4695), anti‐pAKT (Abcam, #Ab81283), anti‐AKT (CST, #4691), anti‐pCDK1/2 (Abcam, #Ab201008), anti‐CDK4 (Abcam, #Ab108357), anti‐PVR (Abcam, #Ab267788).

### RNA Isolation and Real‐Time qPCR Analysis

RNA was extracted from cell lines by using RNA isolation kit according to the manufacturer's instructions (Yeasen, #19221ES). Reverse transcription was performed using the PrimeScriptTM RT Reagent Kit with gDNA Eraser (TaKaRa, #RR047Q). Real‐time qPCR was conducted using SYBR Green qPCR Master Mix (Bimake, #B21702) and a QuantStudio 7 Flex Real‐ Time PCR System (Thermo Fisher). The primer information can be found in Table S1 (Supporting Information).

### IHC Staining

Formalin‐fixed, paraffin‐embedded (FFPE) tumor and paratumor tissues were prepared for tissue microarray as previously reported.^[^ ^13^ ^]^ The slides were baked at 65 °C overnight. After deparaffinization and hydration, these slides were boiled in citrate buffer at 100 °C for 15 min. Subsequently, a 3% H_2_O_2_ solution was used to block endogenous peroxidase activity for 20 min. To prevent nonspecific antibody binding, the slides were then incubated with 5% normal goat serum for 1 h at room temperature. Then these slides were incubated with primary antibodies at 4 °C overnight. Following three washes with TBST, the slides were incubated with an HRP‐conjugated goat anti‐rabbit/mouse secondary antibody (GeneTech, #GK500705) for 1 h at room temperature. The sections were stained with DAB (Vector, #PK‐6100) and then counterstained with hematoxylin according to the manufacturer's instructions. The 3Dhistech Pannoramic Scan system was used for image acquisition. A pathologist performed semiquantitative analysis of IHC staining in a blinded manner by calculating the percentage of positive cells and the positivity degree. Primary antibodies used in the current investigation were listed below: anti‐human‐Plac1 (Novus, #NBP2‐32379), anti‐SP1 (Abcam, #Ab231778), anti‐Ki67 (Servicebio, #GB111141), anti‐mouse‐Plac1 (Absin, #Abs103873), anti‐CD8 (Abcam, #Ab101500), anti‐CD68 (Abcam, #Ab955), anti‐CD11c (Abcam, #Ab52632), and anti‐Foxp3 (Abcam, #Ab215206).

### mIF Staining

mIF staining of 4‐µm FFPE sections was performed using the PANO 4‐plex IHC kit (Absin, #Abs50012) according to the manufacturer's instructions. Different primary antibodies were sequentially applied, followed by horseradish peroxidase‐conjugated secondary antibody incubation and tyramide signal amplification. The glass slides were microwave heat‐treated following each round of TyramideSignal Amplification. Nuclei were stained with DAPI (Sigma, #D9542) after labeling human antigens. The following antibodies were used for mouse samples: anti‐CD4 (Abcam, #Ab183685), anti‐Foxp3 (Abcam, #Ab215206), anti‐CD8 (Abcam, #Ab217344), and anti‐PD1 (Abcam, #Ab214421); and for human samples: anti‐Plac1 (Novus, #NBP2‐32379), anti‐CD4 (Abcam, #Ab133616), anti‐Foxp3 (Abcam, #Ab36607), anti‐CK5 (Abcam, #Ab52635), anti‐PVR (Abcam, #Ab267788), anti‐TIGIT (CST, #99 567), anti‐LTBR (proteintech, #20331‐1‐AP), and anti‐LTA (proteintech, #13111‐1‐AP). The 3Dhistech Pannoramic Scan system was used for image acquisition.

### In Vivo Animal Studies

Subcutaneous tumorigenesis models were established using Balb/c nude mice or C3H/HeJ mice. A total of 1 × 10^6^ cells in 100 µL PBS were inoculated subcutaneously into the right flanks of mice as indicated. The length (L) and width (W) of the tumors were measured every 2 days after injection, and the volume was calculated as (L × W^2^)/2. For the CD4/CD8 depletion assay, αCD4 antibody (Bioxcell, #BE0003) and αCD8 antibody (Bioxcell, #BE0061) were intraperitoneally administrated (200 µg) at days 3, 5, and 8. At the end point of the experiment, mice were sacrificed and tumor tissues were collected for further analysis.

Orthotopic transplants were established as previously reported.^[^ ^73^ ^]^ Mice underwent general anesthesia and were kept on a heating pad. A total of 5 × 10^4^ SCC7 cells in 5 µL PBS (containing 0.5 µL Matrigel matrix gel) were injected into the left side of the tongues of C3H/HeJ mice, and were monitored every 3 days. The mice were euthanized when their body weight was reduced by 20%. For anti‐PD1 treatment experiments, anti‐PD1 (Bioxcell, #BE0146) or isotype IgG (Bioxcell, #BE0089) were intraperitoneally administrated (10 mg kg^−1^) at days 5, 8, and 11.

For the induction of autochthonous mouse HNSCC, mice were consecutively treated by 100 µg mL^−1^ 4‐Nitroquinoline (4NQO, Santa Cruz, #sc‐256815)‐containing drinking water for 16 weeks and then by normal drinking water for another 4–8 weeks. For in vivo genetic deletion of tumor cell *Plac1*, mice were intraperitoneally administered three consecutive injections of 25 mg mL^−1^ tamoxifen (Sigma, #5648). Mice were euthanized at the end point of the experiment and tongue tissues were collected for further analysis.

### TILs Analysis by Flow Cytometry

Tumor single‐cell suspensions of mouse tumors were prepared from mouse tumors using a tumor dissociation kit (Miltenyi, #130‐096‐730). In short, excised tumors were cut into small pieces, enzymatically digested with an enzyme mixture for 25 min at 37 °C, mechanically dissociated, and filtered. The single‐cell suspensions were treated with erythrocyte lysis buffer. The single‐cell suspensions were incubated with Fixable Viability Stain 450 (BD Pharmingen, #562 247), and then stained with anti‐CD45 (Biolegend, #103 107), anti‐CD4 (Biolegend, #100 422), anti‐CD8 (Biolegend, #100 707), anti‐PD1 (Biolegend, #109 120), and anti‐Foxp3 (Biolegend, #126 408). Data were acquired by flow cytometry (BD Biosciences).

### Statistical Analysis

All data were shown as mean ± SD. The details about statistic parameters were specified in the figure legends. All statistical analysis was performed using GraphPad Prism 8 software. Wilcoxon rank‐sum or Student's *t* tests were used to compare two groups. The Kaplan–Meier method and log‐rank test were conducted to evaluate survival differences between two groups. The correlation between the activities of two gene sets was tested by Pearson correlation analysis. Chi‐square test was utilized for comparison of cancer stages in the in‐house TMA cohort and numbers of lesions in the autochthonous mouse HNSCC model. Hypergeometric test was used for GO and KEGG analysis. Empirical shuffling test was used for ligand‐receptor analysis. *P* value of less than 0.05 was considered significant.

## Conflict of Interest

The authors declare no conflict of interest.

## Supporting information



Supporting Information

## Data Availability

The bulk RNA‐seq publicly available data used in this study are available in the TCGA portal (http://gdac.broadinstitute.org/) and the Gene Expression Omnibus under accession code GSE91061.^[^ ^26^ ^]^ Reanalyzed publicly available bulk RNA‐seq data of patients undergoing immunotherapy (Gide2019_PD1 cohort^[^ ^25^ ^]^ and Nathanson2017 cohort^[^ ^27^ ^]^) were downloaded from TIDE database (http://tide.dfci.harvard.edu/),^[^ ^74^ ^]^ and IMvigor210 cohort^[^ ^28^ ^]^ (Intervention treatment of advanced urinary tract transitional cell carcinoma) were downloaded from http://researchpub.gene.com/IMvigor210CoreBiologies/). The processed publicly available scRNA‐seq data used in this study are available in the Gene Expression Omnibus under accession code GSE103322,^[^ ^9^ ^]^ GSE181919,^[^ ^12^ ^]^ GSE185965,^[^ ^19^ ^]^ GSE188737,^[^ ^20^ ^]^ GSE182227,^[^ ^21^ ^]^ GSE173855,^[^ ^22^ ^]^ GSE36552,^[^ ^30^ ^]^ GSE44183,^[^ ^31^ ^]^ and GSE71317. The processed publicly available ST data in this study are available in the Gene Expression Omnibus under accession code GSE208253^[^ ^49^ ^]^ and GSE220978.^[^ ^23^ ^]^ The raw data of scRNA‐seq generated in this study were deposited in Genome Sequence Archive (GSA) with accession ID HRA004648. Since these data are related to human genetic resources, raw data can be obtained directly by requesting and following the GSA guidelines for academic use at https://ngdc.cncb.ac.cn/gsa‐human/browse/HRA004648 after the user log in to the GSA database with the email address of the academic institution. The request will be responded to within two weeks. Once access is granted, users have six months to download the data. The guidance for making a data access request of GSA for humans can be downloaded from https://ngdc.cncb.ac.cn/gsa‐human/document/GSA‐Human_Request_Guide_for_Users_us.pdf.
