# Supplementary material for: Plac1+ Tumor Cell‐Treg Interplay Supports Tumorigenesis and Progression of Head and Neck Cancer
Source: Adv Sci (Weinh). 2025 Mar 8;12(17):2417312. doi: 10.1002/advs.202417312 (PMC12061273; doi:10.1002/advs.202417312)
Supplement: Supplementary file 1 — Supporting Information [file ADVS-12-2417312-s001.pdf]

ADVANCED SCIENCE
Open Access

Supporting Information

for *Adv. Sci.*, DOI 10.1002/advs.202417312

Plac1^{+} Tumor Cell-Treg Interplay Supports Tumorigenesis and Progression of Head and Neck Cancer

*Xiaoyan Meng, Zhonglong Liu, Lu Zhao, Ran Li, Luoman Gan, Liren Cao, Jingjing Sun, Lingfang Zhang and Yue He**

Supporting Information

Plac1^{+} Tumor Cell-Treg Interplay Supports Tumorigenesis and Progression of Head and Neck Cancer

*Xiaoyan Meng, Zhonglong Liu, Lu Zhao, Ran Li, Luoman Gan, Liren Cao, Jingjing Sun, Lingfang Zhang, and Yue He**

X. Y. Meng, Dr. Z. L. Liu, Dr. L. Zhao, Dr. R. Li, L. M. Gan, L. R. Cao, Prof. Y. He
Department of Oral Maxillofacial & Head and Neck Oncology, Shanghai Ninth People's Hospital, Shanghai Jiao Tong University School of Medicine; College of Stomatology, Shanghai Jiao Tong University; National Center for Stomatology; National Clinical Research Center for Oral Diseases; Shanghai Key Laboratory of Stomatology, Shanghai 200011, P.R. China;
E-mail: william5218@126.com

Dr. J. J. Sun
Department of Oral Pathology, Shanghai Ninth People's Hospital, Shanghai Jiao Tong University School of Medicine; College of Stomatology, Shanghai Jiao Tong University; National Center for Stomatology; National Clinical Research Center for Oral Diseases; Shanghai Key Laboratory of Stomatology, Shanghai, 200011, P.R. China;

L. F. Zhang
Suzhou Lingdian Biotechnology Co., Ltd, Suzhou, 215000, P.R. China;

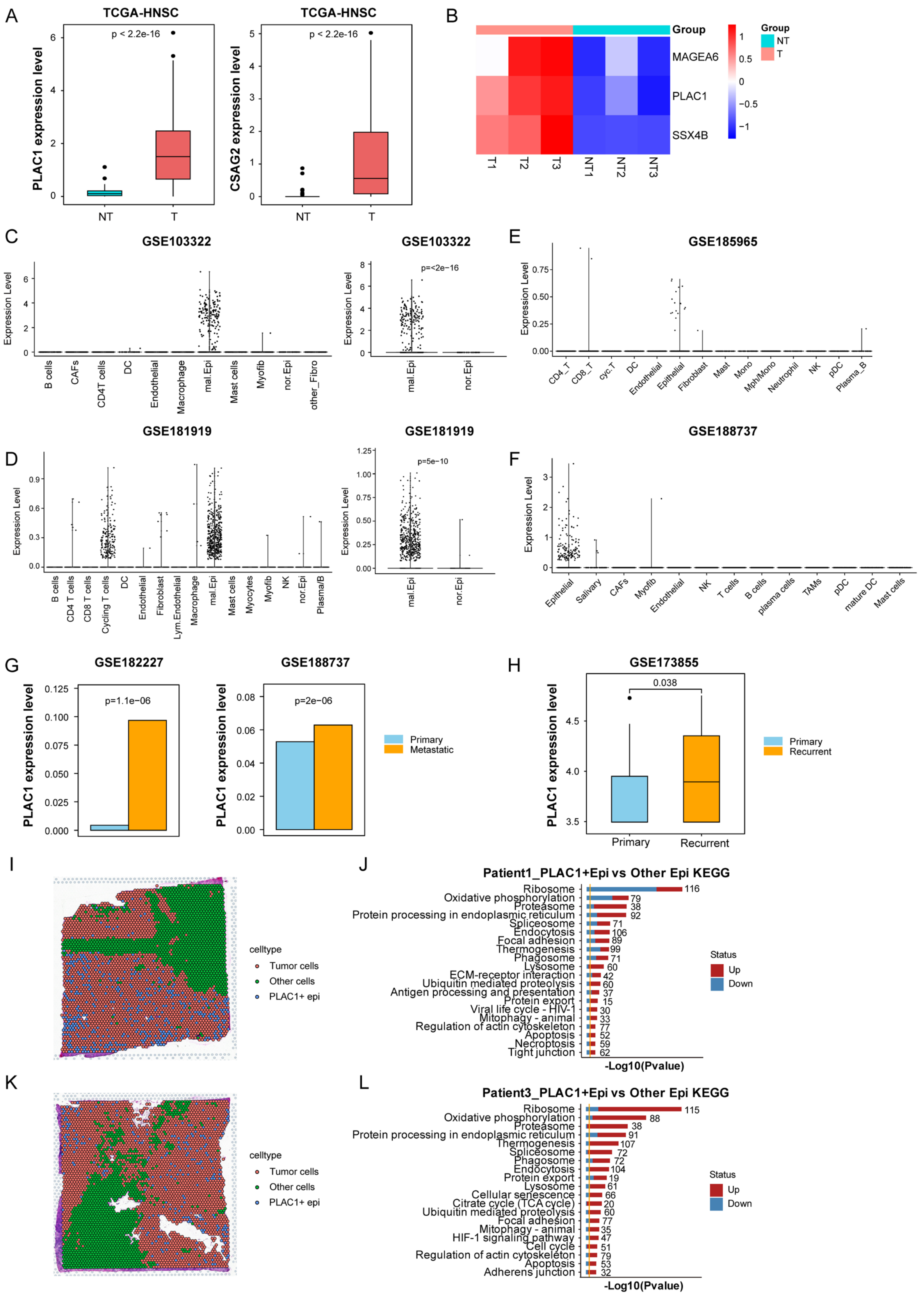

Figure S1 (A) Box plots show *Plac1* (left) and *CSAG2* (right) expression in the TCGA-HNSC cohort. (B) Heatmap shows different gene expression levels in the in-house bulk RNA-seq cohort. (C-D) Violin plots show expression levels of *Plac1* in different cell types from GSE103322 (C), GSE181919 (D), GSE185965 (E), and GSE188737 (F). (G) Bar plots show

different *Plac1* expression levels in epithelial cells of primary and metastatic lesions in GSE182227 (left) and GSE188737 (right). (H) Box plot shows different *Plac1* expression levels of primary and recurrent tumors in GSE173855. (I-L) Distribution pattern of *Plac1*$^+$ epithelial cells (I, K) and KEGG enrichment analysis of DEGs of *Plac1*$^+$ epithelial cells compared to other epithelial cells (J, L). (I-J) show results of patient1 and (K-L) show results of patient3 in GSE220978. *P* values were calculated by two-sided Student's *t*-test in A, C, D, G, H, and by hypergeometric test in J, L.

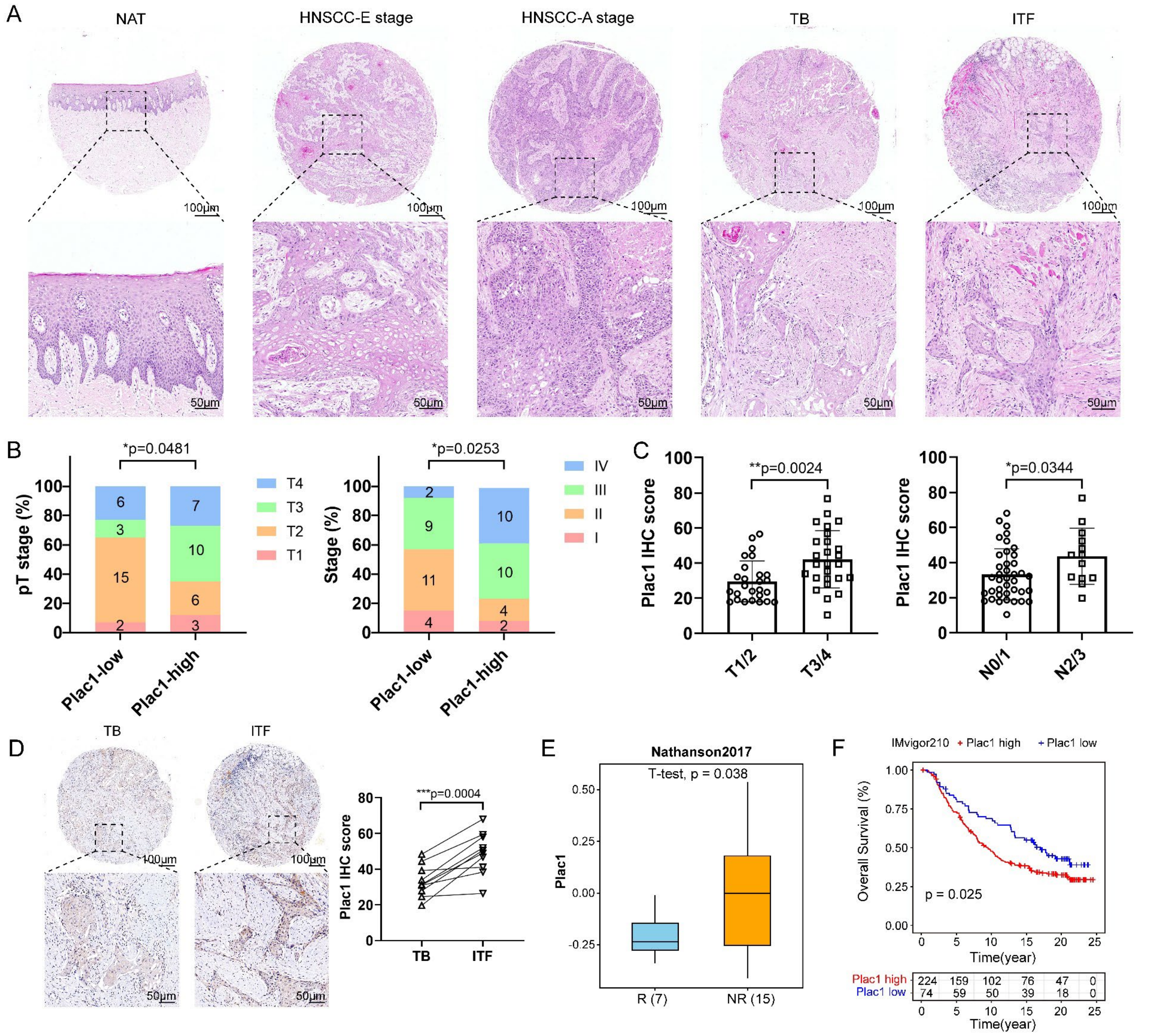

Figure S2 (A) Representative H&E staining of tissue samples during HNSCC initiation and progression. Scale bar, 100 μm and 50 μm. (B) Bar plots show percentage of different pT stages (left) and clinical stages (right) in Plac1-low and -high HNSCC samples (n = 26 for each group). (C) Box plots show Plac1 IHC scores in HNSCC samples of different T stages (left) (T1/2: n = 26, T3/4: n = 26) and N stages (right) (N0/1: n = 39, N2/3: n = 13). (D) Representative IHC staining (left) and quantitative results (right) of Plac1 expression in different HNSCC regions (n = 10). Scale bar, 100 μm and 50 μm. (E) Box plot shows Plac1 expression levels in response and nonresponse groups of Nathanson2017 cohort (n = 22). (F) The Kaplan-Meier curve shows patients with higher Plac1 expression exhibit poorer OS in IMvigor210 cohort (n = 298). *P* values were calculated by unpaired two-sided Student's *t*-test in C, E, by paired two-sided Student's *t*-test in D, by Chi-square test in B, and by two-sided log-rank test in F. *$p < 0.05$, **$p < 0.01$, ***$p < 0.001$.

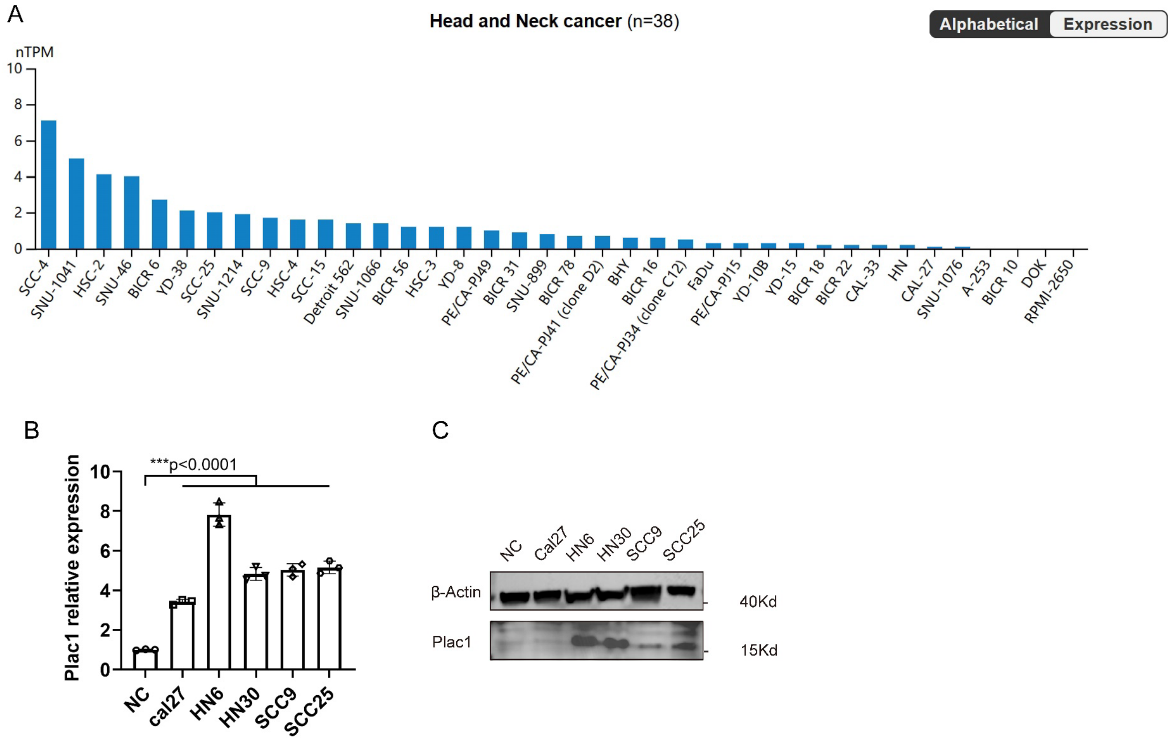

Figure S3 (A) *Plac1* mRNA expression levels in different HNSCC cell lines (from Protein Atlas: https://www.proteinatlas.org/). (B-C) Plac1 mRNA (B) (n = 3) and protein (C) expression levels in normal epithelial cells (NC) and different HNSCC cell lines. *P* values were calculated by two-sided Student's *t*-test in B. $^{***}p < 0.001$.

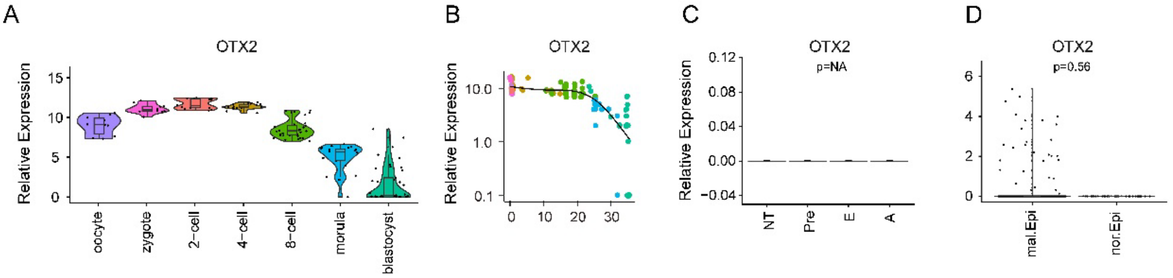

Figure S4 (A-B) The expression level of *OTX2* in different stages during embryonic development (A) and alongside the pseudotime axis (B). (C-D) Violin plots show *OTX2* expression levels in malignant and normal epithelial cells of in-house cohort (C) and GSE103322 (D). *P* values were calculated by one-way ANOVA in C, and by one-sided Wilcoxon test in D.

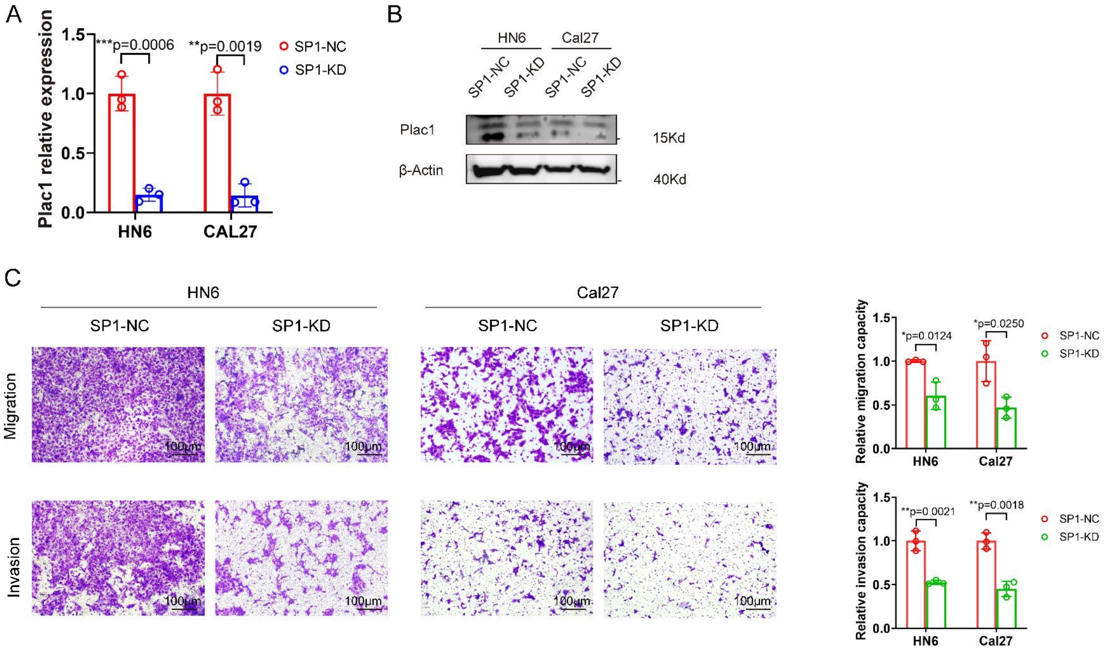

Figure S5 (A-B) Plac1 mRNA (A) (n = 3) and protein (B) expression levels in HNSCC cell lines with or without *SP1* knockdown. (C) Representative images (left) and quantification results (right) of Transwell assay of HNSCC cells with or without *SP1* knockdown (n = 3). Scale bar = 100 μm. *P* values were calculated by two-sided Student's *t*-test in A, C. * $p < 0.05$, ** $p < 0.01$, *** $p < 0.001$.

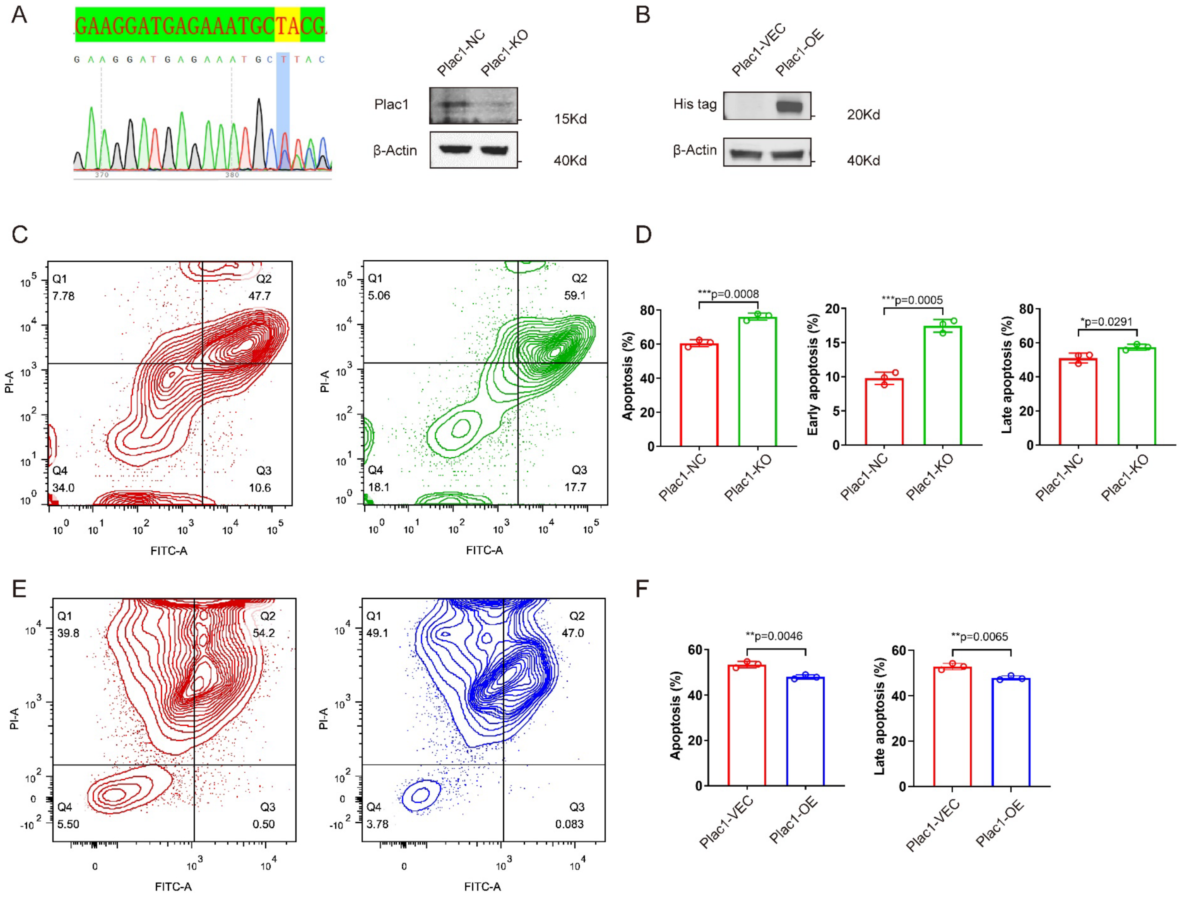

Figure S6 (A) Genotype alignment (left) and WB validation (right) of Plac1-KO HNSCC cell line. (B) WB validation of Plac1-OE HNSCC cell line. (C-F) Representative flow cytometry images (C, E) and quantification results of apoptosis ratio (D, F) of Plac1-KO (C-D) and Plac1-OE (E-F) cells (n = 5). *P* values were calculated by two-sided Student's *t*-test in D, F. $*p < 0.05$, $**p < 0.01$, $***p < 0.001$.

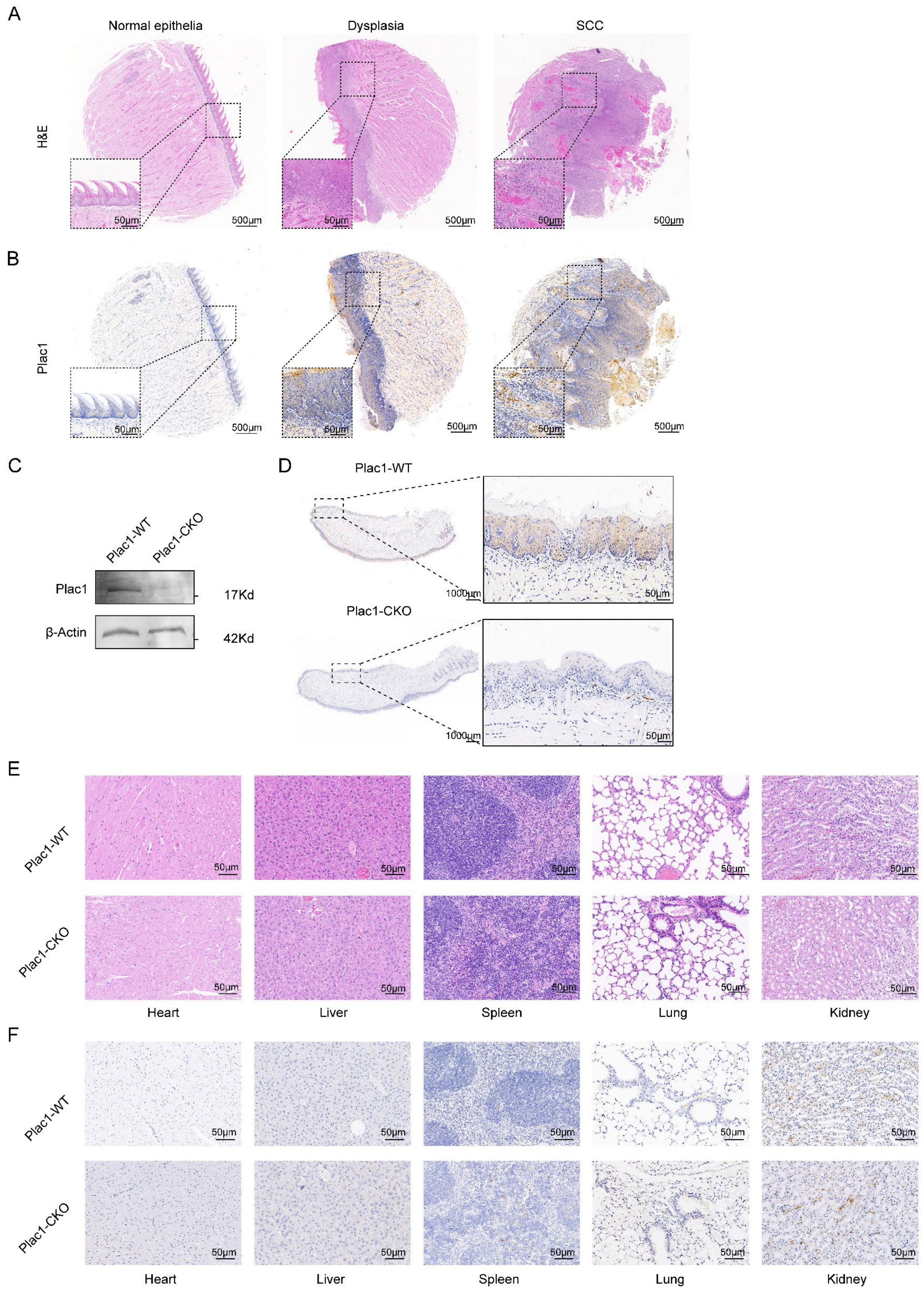

Figure S7 (A-B) Representative H&E staining (A) and Plac1 IHC staining (B) of mice tongue tissues during HNSCC initiation and progression. Scale bar, 500 µm and 50 µm. (C-D) WB (C) and IHC (D) validation of Plac1 deletion in epithelial cells. Scale bar, 1000 µm and 50 µm. (E-F) Representative H&E staining (A) and Plac1 IHC staining (B) of major organs of Plac1-WT and Plac1-CKO mice. Scale bar = 50 µm.

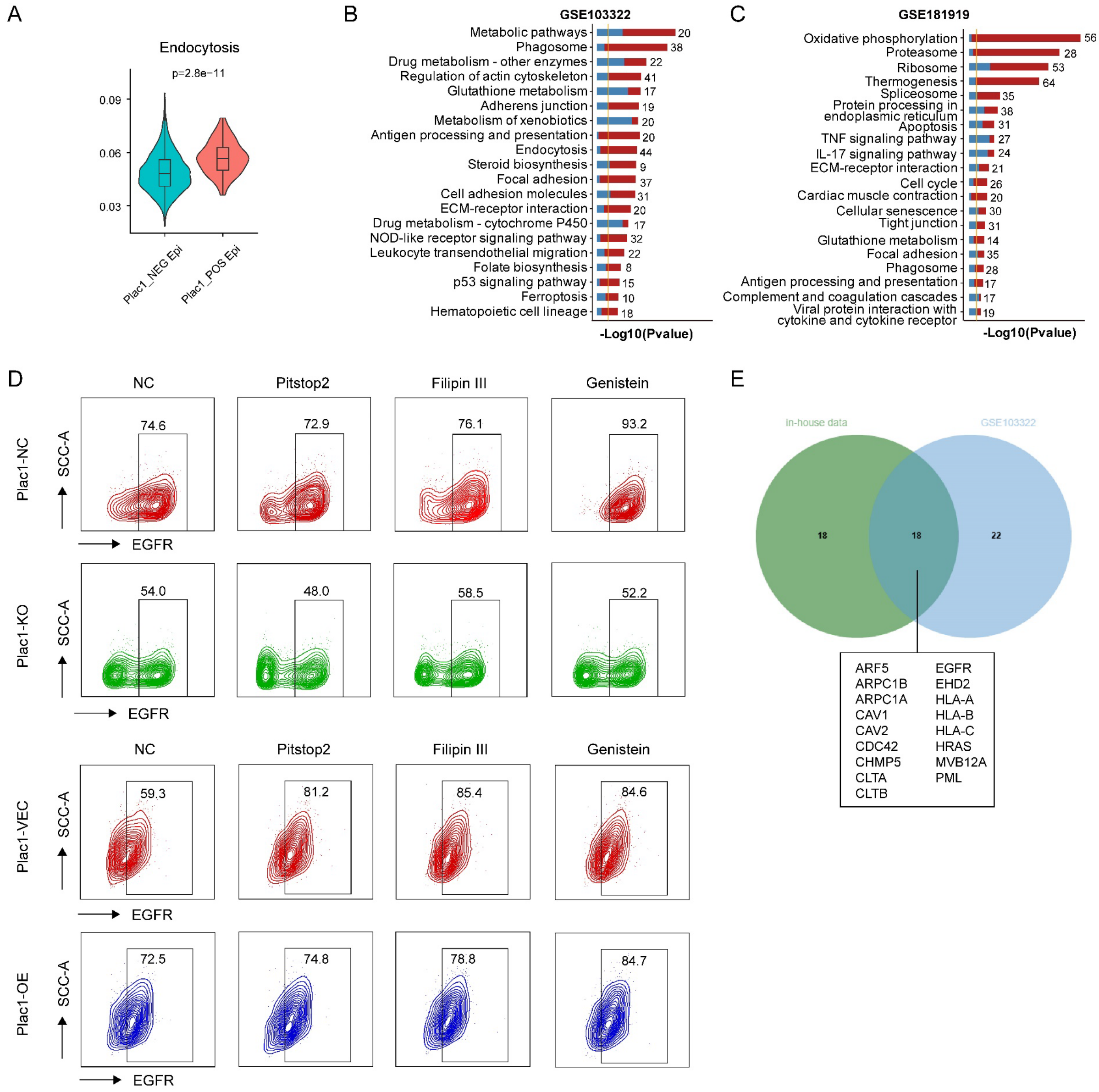

Figure S8 (A) Violin plot shows the expression level of endocytosis signature of *Plac1*$^{+}$ and *Plac1*$^{-}$ malignant epithelial cells of the in-house scRNA-seq cohort. (B-C) KEGG pathway enrichment of DEGs between *Plac1*$^{+}$ and *Plac1*$^{-}$ malignant epithelial cells of GSE103322 (B) and GSE181919 (C). (D) Representative flow cytometry images of HNSCC cells treated with different endocytosis inhibitors. (E) Venn diagram shows the overlapped up-regulated genes (*Plac1*$^{+}$ vs *Plac1*$^{-}$ malignant epithelial cells) related to endocytosis in the in-house scRNA-seq cohort and GSE103322. *P* values were calculated by two-sided Student's *t*-test in A, and by hypergeometric test in B, C.

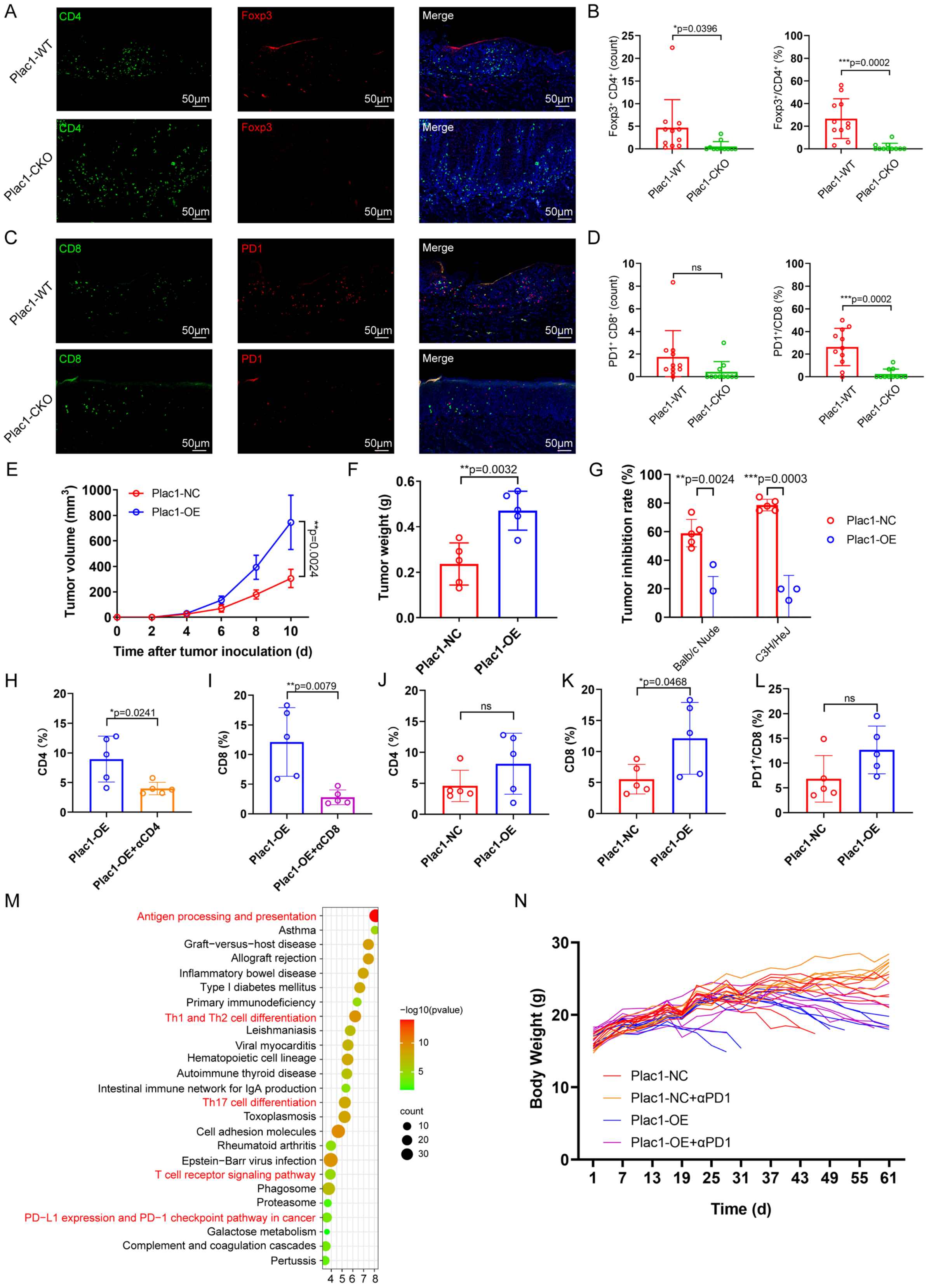

Figure S9 (A) Representative IF images (A, C) and quantification results (B, D) of Tregs (A-B) and CD8_Tex (C-D) of tongue tissues of Plac1-WT and Plac1-CKO mice (n = 11). Green: CD4 (A) or CD8 (C), red: Foxp3 (A) or PD1 (C), blue: Dapi. Scale bar = 50 μm. (E-F) Tumor volumes (E) and tumor weights (F) of mice injected with Plac1-NC and Plac1-OE HNSCC cells

(n = 5). (G) Comparison of tumor inhibition rate of subcutaneous xenograft tumor model in Balb/c Nude mice and C3H/HeJ mice (n = 5). (H-L) Quantification results of different cell ratio of different groups, as indicated (n = 5). (M) GO enrichment of DEGs between tumors in Plac1-NC and Plac1-OE groups. (N) Individual body weight curves of different groups (n = 7). *P* values were calculated by two-sided Student's *t*-test in B, D, E-L. *$p < 0.05$, **$p < 0.01$, ***$p < 0.001$.

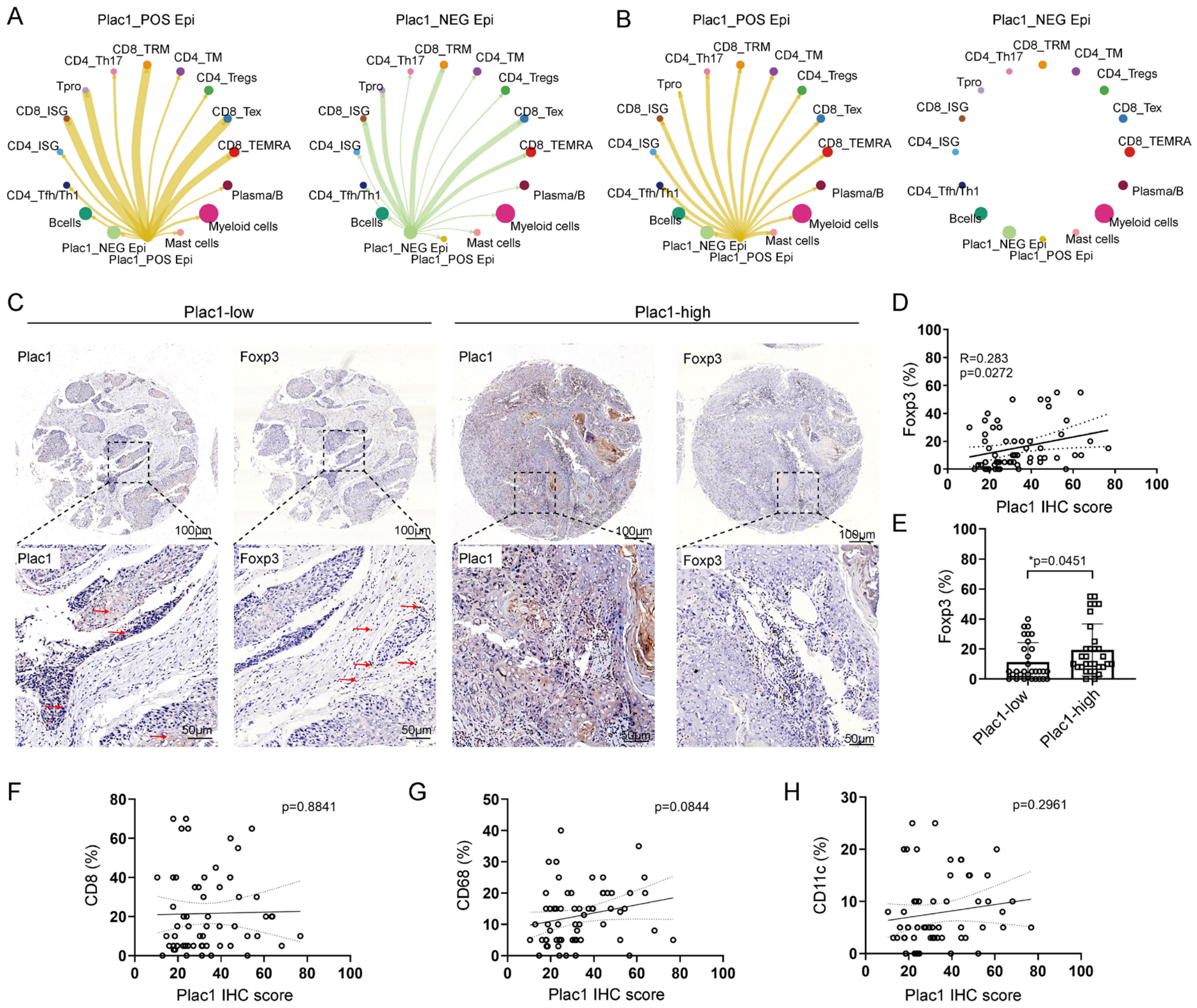

Figure S10 (A-B) Circle plots show the comparison of cell-cell interaction strengths of *Plac1*$^{+}$ and *Plac1*$^{-}$ malignant epithelial cells with other immune cells. (A) shows results of CT and (B) shows results of ER. (C) Representative Plac1 and Foxp3 IHC images of HNSCC tissues. Scale bar, 100 μm and 50 μm. IHC positive areas of Plac1 are cytoplasm of epithelial cells and IHC positive areas of Foxp3 are nucleus of immune cells, as indicated by the red arrows. (D) Pearson correlation of Plac1 IHC score and Foxp3^{+} cell ratio, related to (C) (n = 61). (E) Comparison of Foxp3^{+} cell ratio in Plac1-low and -high HNSCC tissues, related to (C). (F-H) Pearson correlation of Plac1 IHC score and CD8^{+} cell ratio (F) (n = 61), CD68^{+} cell ratio (G) (n = 61), and CD11c^{+} cell ratio (H) (n = 61). *P* values were calculated by two-sided Student's *t*-test in E. *$p < 0.05$.

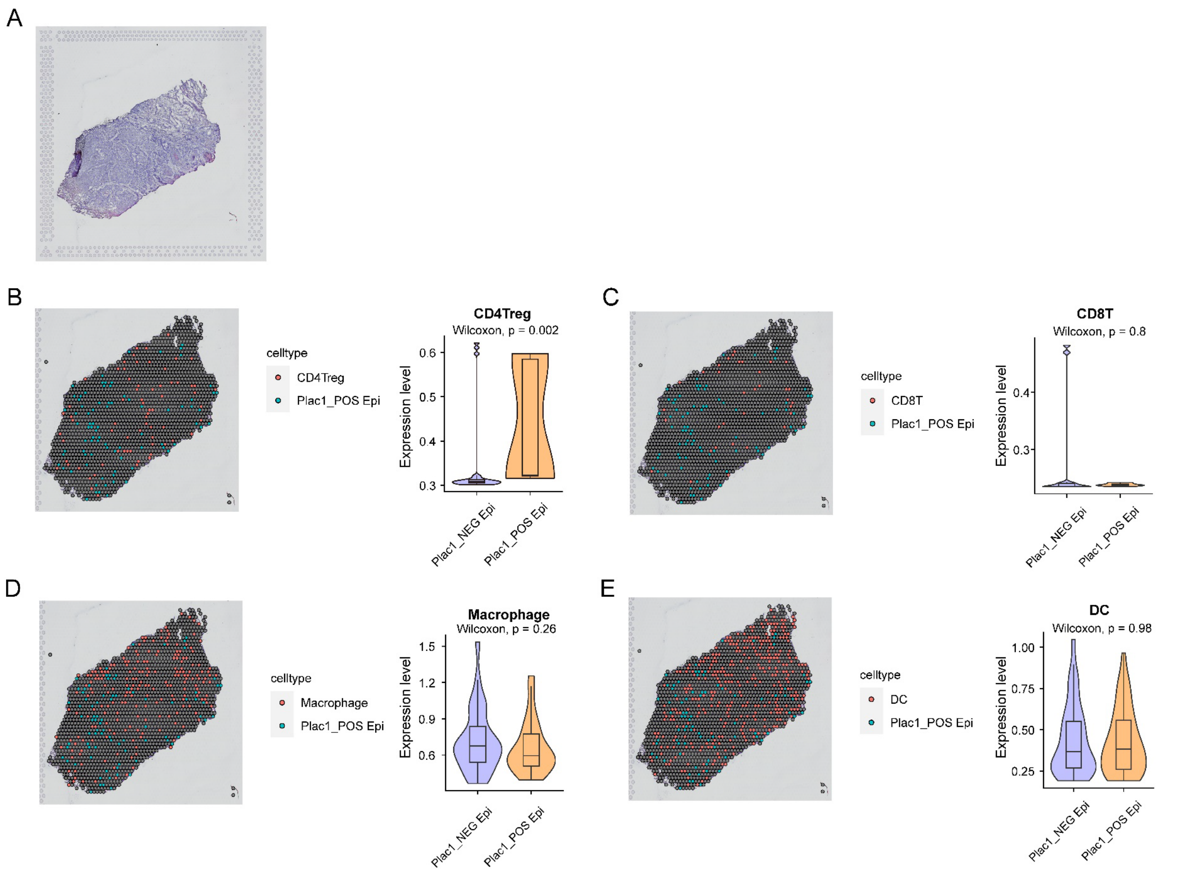

Figure S11 (A) H&E image of HNSCC spatial transcriptome tissue. (B-E) Comparison of expression level of CD4_Treg (B), CD8_T (C), Macrophage (D), and DC (E) of dots around *Plac1*$^{+}$ and *Plac1*$^{-}$ epithelial cells. *P* values were calculated by two-sided Wilcoxon test in B-E.

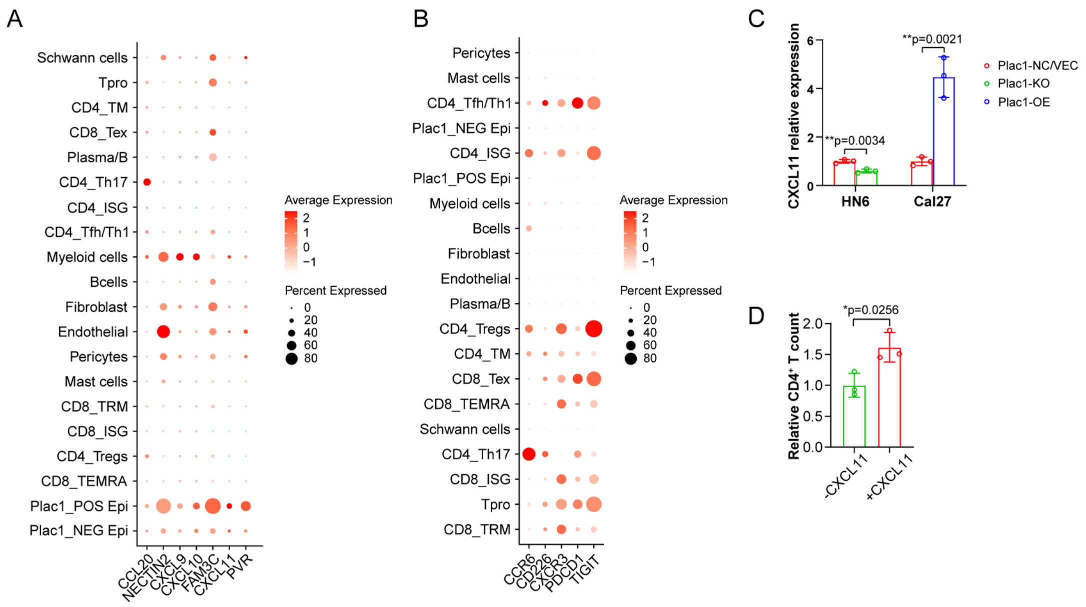

Figure S12 (A-B) Dot plots show expression levels of predicted ligand (A) and receptor (B) molecules in different cell types. (C) mRNA expression level of *CXCL11* in different HNSCC cells (n = 3). (D) Quantification of migrated CD4^{+} T cells after treated with rhCXCL11 (n = 3). *P* values were calculated by two-sided Student's *t*-test in C, D. *$p < 0.05$.

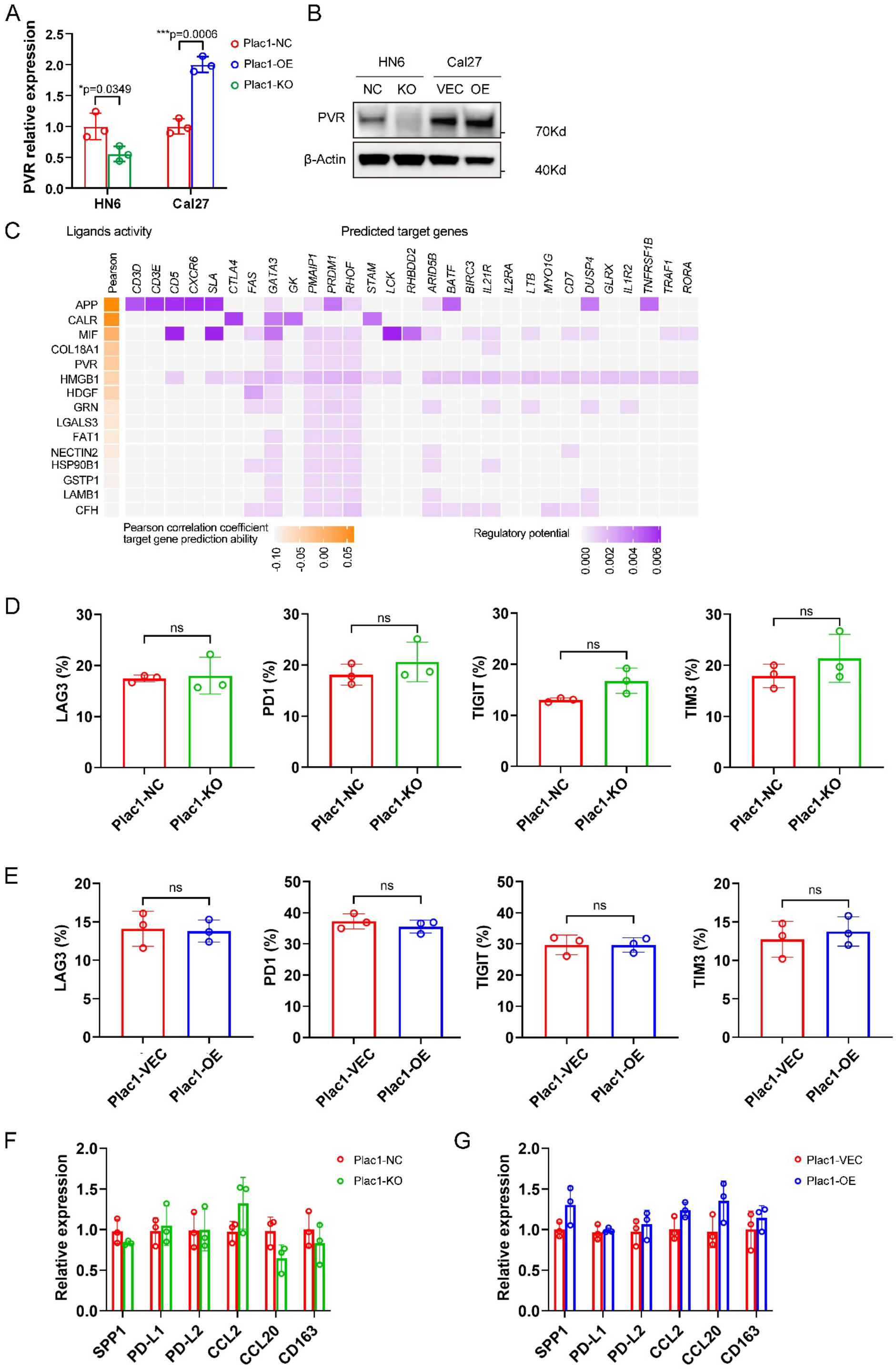

Figure S13 (A-B) mRNA (A) and protein (B) expression level of PVR in different HNSCC cells (n = 3). (C) Heatmap of Nichenet analysis shows regulatory patterns of *Plac1*$^{+}$ malignant epithelial cells to CD4^{+} T cells. (D-E) Quantification of indicated cell ratio of CD8^{+} T cells cocultured with Plac1-NC/KO (D) and Plac1-VEC/OE (E) HNSCC cells (n = 3). (F-G) mRNA expression levels of *SPP1*$^{+}$ macrophage marker genes of macrophages cocultured with Plac1-NC/KO (F) and Plac1-VEC/OE (G) HNSCC cells (n = 3). *P* values were calculated by two-sided Student's *t*-test in A, D-G. *p < 0.05, ***p < 0.001.

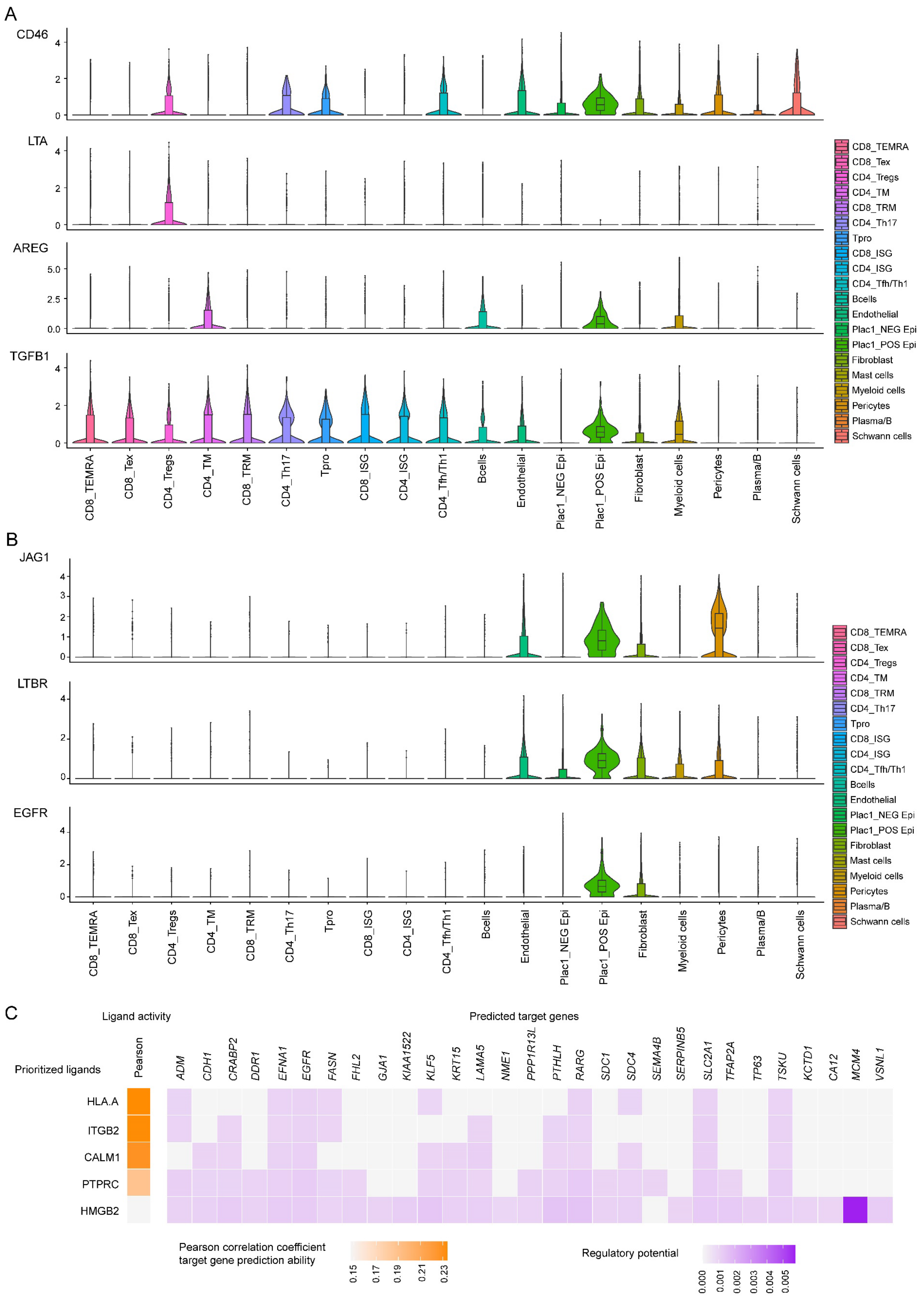

Figure S14 (A-B) Violin plots show expression levels of predicted ligands (A) and receptors (B) in different cell types. (C) Heatmap of Nichenet analysis shows regulatory patterns of Tregs to *Plac1*$^{+}$ malignant epithelial cells.

Figure 4I

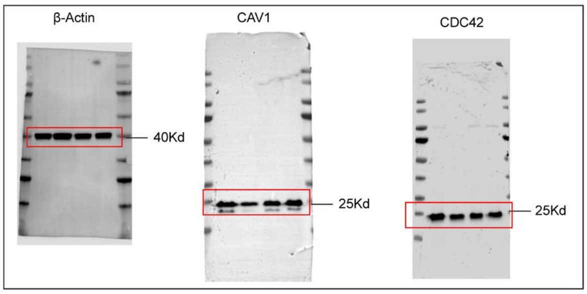

Figure 4J

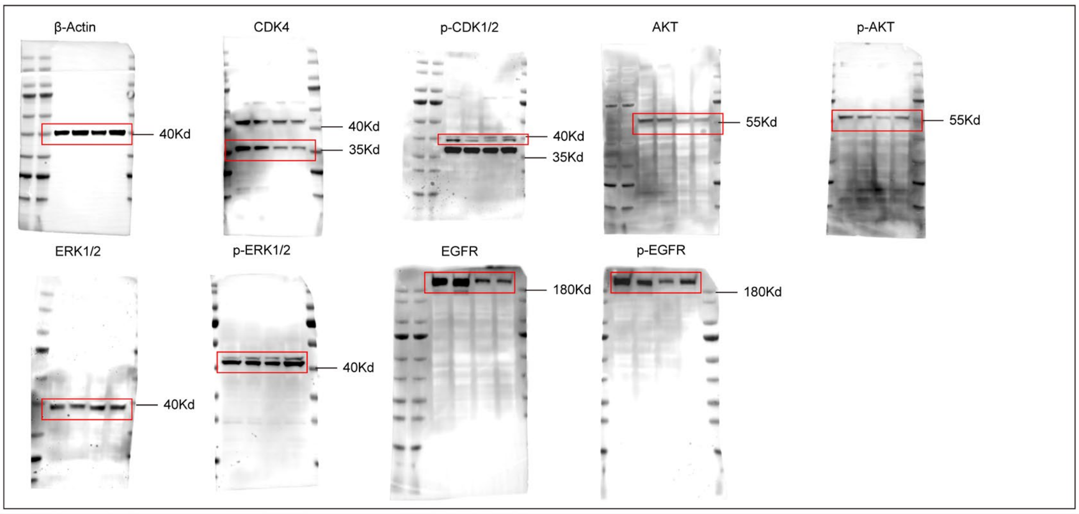

Figure 7G HN6

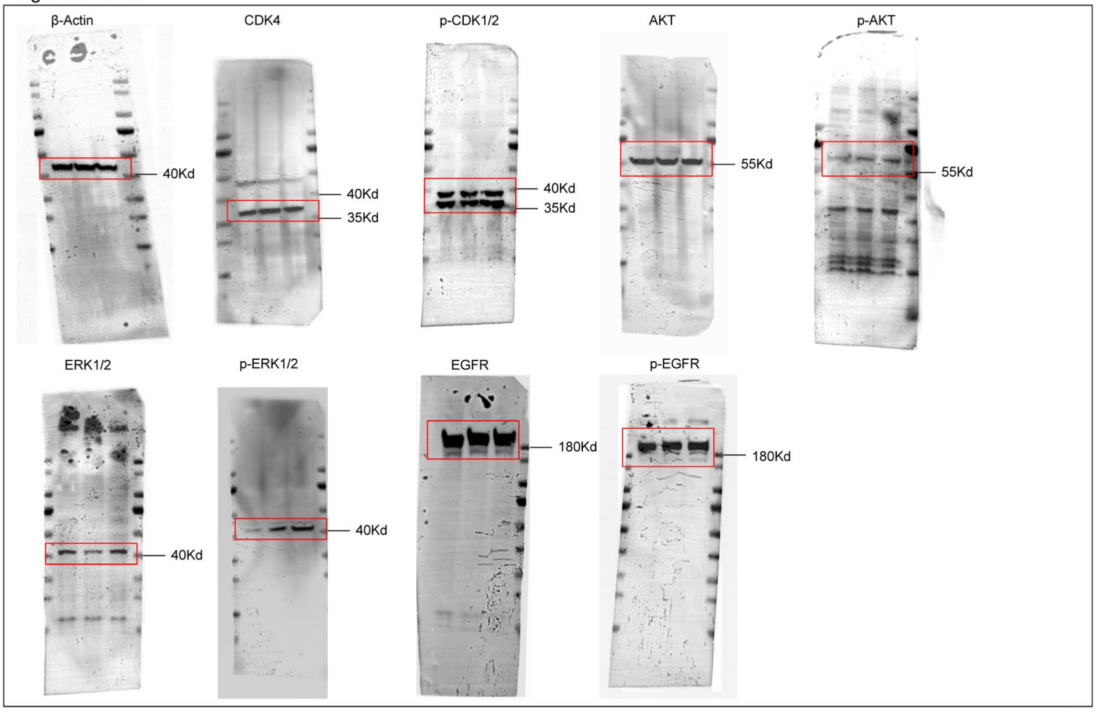

Figure 7G Cal27

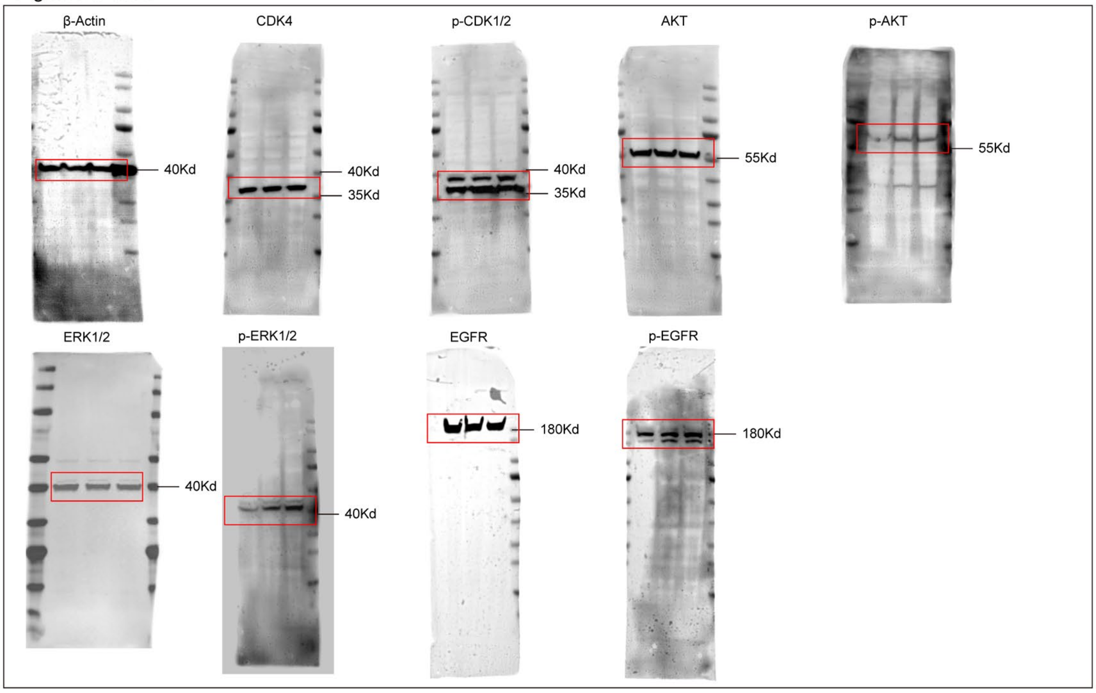

Figure S15 Original images of Western Blotting.

Table S1 Primer information in this study

Primer	F'	R'
hu-Plac1	CAGTGCACCCCTTCATGCTA	GAGACAGCTTTGGCCCTGAT
hu-CAV1	GCATTTACTTCGCCATTCTCTCTTTC	GTAGATGGAATAGACACGGCTGATG
hu-CAV2	CGGCTCAACTCGCATCTCAAG	CAGGAACACCGTCAGGAACTTG
hu-CDC42	GGCTGTCAAGTATGTGGAGTGTTC	TGCGGCTCTTCTTCGGTTCTG
hu-CXCL11	GTGCTACAGTTGTTCAAGGCTTCC	CTGCCACTTTCACTGCTTTTACCC
hu-PVR	GCCCGCCAGCCCAAATCAC	CAGGTCACATTCTTGCCGTCCAC
hu-SPP1	CAGCCGTGGGAAGGACAGTTATG	TCACATCGGAATGCTCATTGCTCTC
hu-PDL1	TGACCTACTGGCATTTGCTGAACG	CACTGCTTGTCCAGATGACTTCGG
hu-PDL2	GCCTCGTTCCACATACCTCAAGTC	GTAGTCCCAGGCGACCCCATAG
hu-CCL2	ACCAGCAGCAAGTGTCCCAAAG	TTTGCTTGTCCAGGTGGTCCATG
hu-CCL20	TACTCCACCTCTGCGGCGAATC	GCATTGATGTCACAGCCTTCATTGG
hu-CD163	GCCACAACAGGTCGCTCATCC	GCAAGCCGCTGTCTCTGTCTTC
hu-CDH1	CTGATTCTGCTGCTCTTGCTGTTTC	GGTCCTCTTCTCCGCCTCCTTC
hu-CDH2	AGGAGTCAGTGAAGGAGTCAGCAG	TTCTGGCAAGTTGATTGGAGGGATG
hu-TWIST1	GTACATCGACTTCCTCTACCAG	CATCCTCCAGACCGAGAAG
hu-SNAIL2	CTGTGACAAGGAATATGTGAGC	CTAATGTGTCCTTGAAGCAACC
hu-VIM	CCTTCGTGAATACCAAGACCTGCTC	AATCCTGCTCTCCTCGCCTTCC
hu-SOX2	CAGCATGTCCTACTCGCAGCAG	CTGGAGTGGGAGGAAGAGGTAACC
hu-SOX9	AAGTCGGTGAAGAACGGGCA	CTGCAGCGCCTTGAAGATGG
hu-MMP2	ATTGTATTTGATGGCATCGCTC	ATTCATTCCCTGCAAAGAACAC
hu-MMP9	CAGTACCGAGAGAAAGCCTATT	CAGGATGTCATAGGTCACGTAG
